# The cobras (genus *Naja*) of Myanmar: An updated species list with information on identification, distributions, and medical importance

**DOI:** 10.1371/journal.pntd.0014445

**Published:** 2026-06-29

**Authors:** Neil R. Balchan, Win Paing Oo, Guinevere O. U. Wogan, Daniel E. Keyler, Wolfgang Wüster

**Affiliations:** 1 Department of Biology, Oklahoma State University, Stillwater, Oklahoma, United States of America; 2 College of Sustainability and Tourism, Ritsumeikan Asia Pacific University, Beppu, Oita, Japan; 3 Native Species Conservation and Identification in Myanmar, Mandalay, Myanmar; 4 Department of Experimental and Clinical Pharmacology, College of Pharmacy, University of Minnesota Twin Cities, Minneapolis, Minnesota, United States of America; 5 MEEB, School of Environmental and Natural Sciences, Bangor University, Bangor, Wales, United Kingdom; Fundação de Medicina Tropical Doutor Heitor Vieira Dourado: Fundacao de Medicina Tropical Doutor Heitor Vieira Dourado, BRAZIL

## Abstract

The cobra diversity of Myanmar has long been a source of confusion, complicating public health responses to snakebites resulting from this medically important genus. By integrating distributional data, natural history information, and clinical evidence, we clarify the composition and distribution of Myanmar’s cobra fauna and evaluate the implications for envenoming and snakebite management. We confirm the presence of five cobra species (*Naja fuxi*, *N. kaouthia*, *N. mandalayensis*, *N. siamensis*, and *N. sumatrana*) in Myanmar through voucher specimens and/or diagnostic photographic records and identify one additional unconfirmed species (*N. sagittifera*) that may occur in the country based on distributional proximity. Encounter records showed significant within-year temporal variation, with reports peaking in December and during the dry season. Because data derive from opportunistic citizen science submissions, these patterns likely reflect seasonal differences in detection and human activity rather than biological seasonality. Differences from wet-season peaks reported elsewhere highlight the importance of sampling framework in shaping apparent temporal trends. Review of clinical and toxicological information shows that only one species is represented in locally produced antivenom, raising concern about limited cross-neutralization for other cobra species in the country. Traditional practices remain common in many communities and include harmful methods that delay access to medical care or worsen injuries. Clinical evidence demonstrates that neurotoxicity, respiratory failure, and localized tissue destruction are the principal complications of cobra envenoming, often requiring antivenom therapy, airway support, mechanical ventilation, infection management, and surgical intervention in severe cases. The broader-than-recognized diversity of cobras in Myanmar, combined with high encounter probabilities, synanthropic tendencies, and gaps in public awareness, continues to hinder effective management of snakebite. Improved community education, expanded venom and antivenom research, and timely access to appropriate medical care are essential for reducing the burden of cobra envenomation in Myanmar.

## Author summary

The cobra fauna of Myanmar has long been poorly understood, with previous studies underrepresenting the diversity of species present in the country. Given the medical importance of cobras and recent advances in taxonomic and evolutionary research within this genus, establishing an accurate taxonomic and distributional framework for cobra diversity in Myanmar is a critical first step. We compiled cobra occurrence records from citizen science platforms and natural history collections and mapped these data to elucidate distributional patterns of cobra species within and adjacent to Myanmar. We present species accounts for five confirmed and one unconfirmed cobra species, summarizing their distributions, natural history, and medical importance. Using observational data, we examine temporal patterns of reported human–cobra encounters and identify barriers to effective snakebite treatment. Finally, we provide an updated overview of the medical management of cobra envenoming across species and emphasize the urgent need for continued research on Myanmar’s cobras and their venoms.

## Introduction

**Fig 1:**
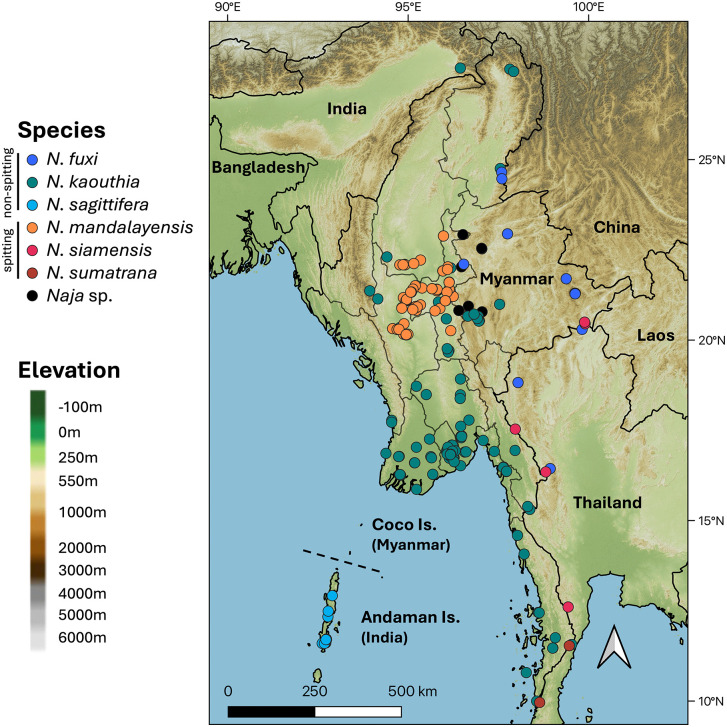
Map of cobra records for all species known from within or near Myanmar. Note that both Myanmar and extralimital records are included (S1 Appendix). Records not assignable to species are listed as *Naja* sp., and dashed line demarcates the division between Coco Islands, Myanmar, and Andaman Islands, India. Base map derived from SRTM 30 arc-second DEM (NASA/ USGS, https://earthexplorer.usgs.gov, accessed 25 January 2025) with political boundaries from the QGIS sample world map (https://qgis.org).

Myanmar is the largest country in mainland Southeast Asia by land area, and it shares borders with China to the northeast, Laos to the east, Thailand to the southeast, Bangladesh to the west, and India to the northwest (Fig 1). The country holds remarkable physiographic diversity, encompassing approximately 15 ecoregions spanning major elevational gradients [1,2], and occupies a biogeographic crossroads where faunal assemblages from China, Indochina, India, the Himalayas, and the Malay Peninsula converge [3]. Owing to its expansive area and broad range of habitats, Myanmar supports a highly diverse reptile fauna, with new species and distributional records continuing to expand the national checklist [3,4]. Of 212 reptile species assessed in Myanmar, approximately 20% are endemic, and 10% are considered microendemics, with 25% of species listed as threatened or potentially threatened according to the IUCN Red List [5]. While the country’s reptile diversity remains understudied, considerable attention has been directed toward Myanmar’s venomous snakes, due to their medical importance [1]. At least 39 venomous snake species are currently known from Myanmar [1] with 19 species designated as medically relevant by the World Health Organization [6], although this number is likely a considerable underestimate pending further taxonomic work [7,8] and updated distributional data [9].

In Myanmar, the only snake antivenoms currently in use are produced by Burmese Pharmaceutical Industries, which manufactures two monovalent antivenom products [10]: one raised against the Siamese Russell’s viper (*Daboia siamensis*; BPI Viper Antivenom) and the other against the monocled cobra (*Naja kaouthia*; BPI Cobra Antivenom). Data from Mandalay General Hospital indicate substantially greater usage of the BPI Viper Antivenom (8,848 vials) compared to the BPI Cobra Antivenom (30 vials) over the twelve-month study period [10], although these figures are unlikely to represent antivenom usage patterns across the country as a whole. Several studies have reported limited cross-neutralization of cobra venoms when treated with antivenoms raised against heterospecifics [11] or conspecifics from geographically distant populations [11–13], although other studies find partial or effective neutralization despite venom–antivenom mismatch [14]. Earlier reference material on Myanmar’s venomous snakes [1] advises against the use of *Naja kaouthia* antivenom in cases of *Naja mandalayensis* envenoming, citing concerns over inadequate cross-neutralization. Given that the BPI cobra antivenom is produced solely with *N. kaouthia* venom, its effectiveness in treating envenoming from other *Naja* species present in Myanmar remains a major concern.

Early assessments of Myanmar’s snake fauna recognized only a single cobra species in the country, identified as *Naja kaouthia* [1]. However, the possibility of additional cobra diversity has long been suggested, with historic reports noting the presence of at least one spitting form alongside the widespread non-spitting type [15]. Subsequent multivariate morphometric analyses confirmed the presence of at least two distinct forms in Myanmar: the widespread non-spitting *N. kaouthia* and a range-restricted, endemic spitting taxon from the region of “Rangoon or Mandalay,” which was later described as a distinct species, *Naja mandalayensis* [16–18]. Most scientific and medical literature has since recognized these two species as comprising the cobra fauna of Myanmar [1,10]. Nevertheless, the likely occurrence of *Naja siamensis* has been suggested, despite a lack of confirmed voucher specimens or credible records [19], and a recent country-wide assessment of Burmese reptiles lists *N. siamensis* as a native species without elaborating further on its distribution [5]. The taxonomy and distributions of Asiatic cobras have been further complicated by the recent description of *Naja fuxi*, a morphologically similar species to *N. kaouthia*, which is now known from southern China [20] and throughout extensive areas of Indochina [21]. The World Health Organization’s Snakebite Information and Data Platform has, since at least 2023, recognized *Naja kaouthia*, *Naja mandalayensis*, *Naja siamensis*, and *Naja sumatrana* in the county, based on distributional maps representing current expert consensus and available data [6], though vouchered records for the latter two species remain unknown. Consequently, the actual number of cobra species present in Myanmar, as well as their geographic distributions, remains poorly resolved.

Currently, only two cobra species, *Naja kaouthia* and *Naja mandalayensis*, are widely recognized as occurring in Myanmar [1,22,23]. However, given the medical importance of this genus and recent taxonomic developments, a more comprehensive assessment of cobra diversity in the country is warranted. In this study, we review *Naja* species with confirmed and potential occurrence in Myanmar based on natural history specimens and citizen science records. We increase the number of confirmed *Naja* species in the country to five and propose the potential occurrence of one additional species. We also summarize current knowledge on the distributions, natural histories, and medical relevance of cobras in Myanmar.

## Methods

### Point data collection

To compile occurrence data for Myanmar cobras, we collected citizen science records from the “Native Species Conservation and Identification in Myanmar” and “Myanmar Snakes” Facebook groups. On 23 December 2025, we queried these Facebook groups using the search terms “*Naja*” and “cobra,” retaining all posts that included verifiable photographic records of cobras. Each record was reviewed and vetted by the authors (NRB and WPO), with date of observation and location information confirmed, often through directly contacting the original observer. Coordinates for all citizen science records were obtained through manual georeferencing of locality data using Google Earth, and no locality points were further obscured or generalized. A total of 138 Facebook records (104 from Native Species Conservation and Identification in Myanmar and 34 from Myanmar Snakes) were assigned to the following taxa based on visible morphological/phenotypic characters (i.e., scalation, hood marks, body pattern): *Naja fuxi* (n = 2), *N. kaouthia* (n = 84), *N. mandalayensis* (n = 37), *N. siamensis* (n = 2), and unidentified *Naja* sp. (n = 13). An additional 32 records were obtained from iNaturalist on 23 August 2025 encompassing *N. fuxi* (n = 9), *N. kaouthia* (n = 11), *N. mandalayensis* (n = 4), *N. sagittifera* (n = 4), *N. siamensis* (n = 3), and *N. sumatrana* (n = 1) occurring within or near the border of Myanmar. All iNaturalist records were reviewed by the authors (NRB and WPO) and contained photographs from which species identifications could be verified. Geographic data were also included for specimens of *N. kaouthia* (n = 21) and *N. mandalayensis* (n = 30) held in the collections of the California Academy of Sciences, and six additional points for *N. sagittifera* were included from a previously published study [24] to better approximate ranges for these three species via a greater number of occurrence points. A single photographic record of *N. sumatrana* was provided to us by local observers, and associated photographs were deposited at the University of Texas at Arlington Amphibian and Reptile Diversity Research Center Digital Collection. All point data (n = 228; S1 Appendix) are plotted on a map of Myanmar (Fig 1) to visualize the distributions of cobras within and adjacent to the country.

### Species accounts

We provide accounts for six *Naja* species with confirmed (voucher specimens or diagnostic photograph with traceable provenance) or potential (unconfirmed/inferred) occurrence in Myanmar: *Naja fuxi*, *N. kaouthia*, *N. mandalayensis*, *N. sagittifera*, *N. siamensis*, and *N. sumatrana*. Each account includes a summary of information from the literature regarding distribution, taxonomy, natural history, and medical importance. Due to the limited research focused specifically on cobras in Myanmar, many of these accounts draw on data from populations outside of the country, however, we incorporate records and information from Myanmar wherever available. Photographs from within or near Myanmar included for each species represent a typical looking adult individual.

### Human-cobra encounters

To explore seasonal patterns in human–cobra interactions, we analyzed a subset of occurrence records (“Native Species Conservation and Identification in Myanmar” and “Myanmar Snakes” Facebook groups and iNaturalist) for *Naja kaouthia* (n = 91) and *Naja mandalayensis* (n = 41), the only two species with sufficient data to plot temporal trends. Duplicate records (e.g., the same image posted to both Facebook and iNaturalist) were combined to avoid overcounting individual encounters, voucher specimens were omitted to avoid biases associated with intentionally collected snakes, and ambiguous records (identified as *Naja* sp.) were not included. Monthly average precipitation data for Yangon were obtained from www.weather-atlas.com and plotted alongside the observation data to visualize potential correlations between rainfall and reported cobra encounters. Monthly and seasonal variation in encounter frequency was evaluated separately for *Naja kaouthia* and *Naja mandalayensis* using chi-square goodness-of-fit tests. For monthly analyses, observed encounter frequencies (January–December) were compared against a null expectation of uniform distribution across 12 months (expected frequency = total annual encounters/ 12). For seasonal analyses, months were grouped into three seasons: dry season (November–February; 4 months), hot season (March–May; 3 months) and rainy season (June–October; 5 months). Because seasons were unequal in duration, expected frequencies were adjusted proportionally to season length (4/12, 3/12, and 5/12 of annual encounters, respectively). To assess whether seasonal distributions differed between species, a chi-square test of independence was performed using a 2 × 3 contingency table (species × season). All tests used α = 0.05.

## Results and discussion

We confirm the presence of five species of cobra in Myanmar based on the presence of voucher specimens and/or diagnostic photographic records (Table 1; Fig 1), the brown-banded cobra (*N. fuxi*), monocled cobra (*N. kaouthia*), Burmese spitting cobra (*N. mandalayensis*), Indochinese spitting cobra (*N. siamensis*), and the equatorial spitting cobra (*N. sumatrana*). We also suggest an additional unconfirmed species may be present in Myanmar, the Andaman cobra (*N. sagittifera*) in the Coco Islands (Table 1; Fig 1). Distributional data, natural history information, and medical importance are summarized for each species below.

**Table 1:** Simplified key to the confirmed (voucher specimens and/or diagnostic photographic records) and possible (inferred) cobra fauna of Myanmar, describing typical appearance for each species at adulthood in or near Myanmar. Note that most species of cobra show high geographic variation, and the provided key is not meant to be exhaustive. Most encounters with cobras in Myanmar involve *Naja* *kaouthia* or *N*. *mandalayensis.*

Species	*Naja fuxi*	*N. kaouthia*	*N. mandalayensis*	*N. sagittifera*	*N. siamensis*	*N. sumatrana*
**Distribution**	Widespread (high elevation)	Widespread (low elevation)	Central Dry Zone	Coco Islands? (unconfirmed)	Divisions bordering Thailand	Southern Tanintharyi Region
**Spitting?**	No (Rare)	No (Rare)	Yes	No (Rare)	Yes	Yes
**Maximum Total Length**	~1.8 m [21]	~2.3 m [25]	~1.2 m [18]	~1.5 m [25]	~1.6 m [19]	~1.5 m [26]
**Hood Mark**	O-shaped or absent	O-shaped or absent	Faint U-shaped or absent	O-shaped	U or V-shaped, or absent	Absent
**Color**	Dark brown	Light to dark brown	Dark greyish-brown	Dark grey to black	Olive to light brown, blackish, or dark and light banded	Black, or yellow-light brown
**Pattern**	Light bands on posterior body, may be faded in adults	Often patternless, some individuals with messy banding	Faint dark crossbanding on some individuals	Dark crossbanding	Variable, often patternless	Patternless or with widely spaced light bands
**# Ventral Scales**	178-201	170-197	173-185	175-183	153-174	179-201

## Species accounts

### Historically documented species

#### *Naja kaouthia* Lesson, 1831 – Monocled cobra.

**Fig 2:**
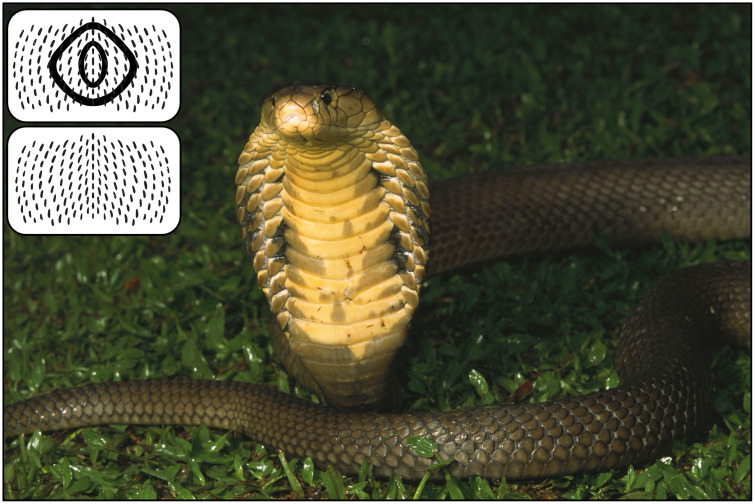
*Naja kaouthia* photographed in Ayeyarwady Division, Myanmar in 2000 by Hla Tun (Myanmar Herpetological Survey), used with permission under CC BY 4.0. Hood marks may be O-shaped or absent (illustrated, top left), and the photographed individual shows a patternless brown body typical of adults of this species.

Distribution – India (eastern and northeastern), Bangladesh, Nepal, Bhutan, China (Yunnan Province), Thailand, Cambodia, Laos, Vietnam (southern), and Malaysia (Peninsular Malaysia, introduced to Sarawak). In Myanmar, *N. kaouthia* is distributed throughout the country in Ayeyarwady (Fig 2), Bago, Chin, Kachin, Kayin, Magway, Mandalay, Mon, Naypyidaw, Rakhine, Sagaing, Shan, Tanintharyi, and Yangon divisions, but it is largely absent in the Central Dry Zone encompassing all of Mandalay and parts of Magway and Sagaing [1,18,27]**.**

Description – Maximum reported adult total length of 2300 mm [25], though total lengths between 1000–1900 mm are more typical [26]. Coloration and pattern are variable both geographically and within populations [27], the dorsal surface typically being brown, yellow, grey, or black and may have irregular or clearly defined crossbanding [26]. The hood mark is generally an O-shaped monocle marking or mask shaped, but may be absent, connected to the ventral scales on one of both sides, or broken on anterior, posterior, or both ends [27].

Natural History – Most active at dusk and night but may also be active during the daytime [26]. Often encountered in villages, agricultural areas, grasslands, coastal rainforests, moist lowlands, and mesic areas, and frequently seeks refuge in holes and termite mounds [1,26]. The monocled cobra preys on mammals, birds, bird eggs, reptiles, amphibians, fish, carrion, and arthropods, with rodents – particularly mice (*Mus* spp.), rats (*Rattus* spp.) and bandicoot rats (*Bandicota* spp.) – being important prey species [28]. This cobra has been observed eating fish caught in fish traps in the Moyingya Wetlands Bird Sanctuary in Bago Division, Myanmar [29]. In Thailand, breeding occurs from August to January, clutches of 10–37 eggs are laid between October to March and hatch between December and May following a 51–69 day incubation period [26]. Neonates weigh 13.2–18.8 g and measure 315–355 mm in length upon hatching [30].

Medical Importance – *Naja kaouthia* is a major contributor to snakebite across its broad Asian range and is classified as a Category 1 medically important species in Myanmar [31]. In the Mandalay region, however, it is responsible for only a minority of cobra envenomings, accounting for three of 18 reported cases in one study [32]. This species shows considerable venom variation across its range [27,33], and antivenoms produced using geographically mismatched venoms may have limited cross-neutralization efficacy [34].

*Naja kaouthia* is considered to be a non-spitting cobra; however, venom spitting is well known from South Asian populations [27], with confirmed records from India [35,36], Nepal [37], and Bangladesh [38]. Although venom spitting has not been reported from Myanmar, its occurrence, particularly in western populations, cannot be ruled out. Venom ophthalmia resulting from *N. kaouthia* can lead to severe symptoms, including intense pain, burning sensations, conjunctival redness, photophobia, scleral hemorrhage, and temporary visual impairment [35,37–39]. Management of *N. kaouthia* venom ophthalmia should follow standard protocols for spitting cobras, including immediate and thorough irrigation of the affected eyes with copious amounts of clean water, followed by prompt medical evaluation [40,41].

#### *Naja mandalayensis* Slowinski and Wüster, 2000 – Burmese spitting cobra.

**Fig 3:**
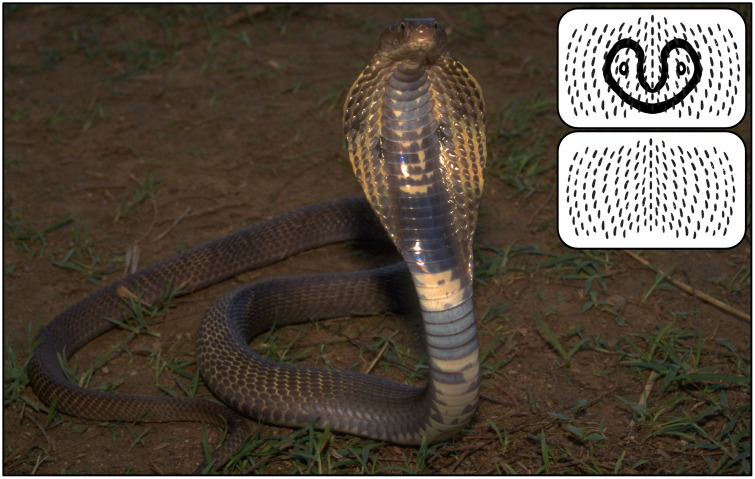
*Naja mandalayensis* photographed in Mandalay Region, Myanmar between 1998–2000 by Hla Tun (Myanmar Herpetological Survey), used with permission under CC BY 4.0. Hood marks may be U- or spectacle shaped or absent (illustrated, top right), and the photographed individual shows dark greyish-brown body coloration with faint cross banding typical of adults of this species.

Distribution – Endemic to Myanmar, *N. mandalayensis* is known only from the Central Dry Zone in parts of Magway, Mandalay (Fig 3), and Sagaing divisions [1,18].

Description – Maximum reported adult snout-vent length of 1018 mm from an individual with a broken tail, total length estimated to be ca. 1268 mm [18]. Little variation among individual patterns, with body coloration uniformly dark greyish-brown and light yellowish-cream interstitial skin, faint dark crossbanding visible on some individuals, ventral side of hood dark brown, with dark throat band present on ventral surface below hood, lateral throat spots on ventral surface of hood lacking. U-shaped or spectacle shaped hood marks often present but may be faint in appearance and confined to the interstitial skin [18].

Natural History – Habitat includes acacia savannahs and stunted dipterocarp savannahs of the Central Dry Zone of Myanmar, but thrives in agricultural areas and around villages, and is frequently encountered in and around human settlements [1,18]. Little is known about the diet of this species, but it is known to consume frogs, snakes, and rodents. Confirmed prey species include kukri snakes (*Oligodon* sp.; S1 Appendix, record #182) and Asian common toad (*Duttaphrynus melanostictus*; S1 Appendix, record #183).

Medical Importance – *Naja mandalayensis* is classified as a Category 1 medically important snake in Myanmar by the World Health Organization [31] and is the primary species responsible for cobra envenomation in the Mandalay region, accounting for 15 of 18 reported cases [32]. The venom of *N. mandalayensis* is compositionally distinct from that of *N. kaouthia*, particularly in its higher relative abundance of cardiotoxic three-finger toxins, although most toxin families are shared between the two species [42]. Despite these compositional differences, BPI Cobra Antivenom has been shown to neutralize *N. mandalayensis* venom with efficacy comparable to that against *N. kaouthia* and is recommended given the absence of a species-specific antivenom [14]. However, substantially higher doses of antivenom than the standard initial dose of 4 vials (recommended for *N. kaouthia* envenoming) may be required for effective treatment of *N. mandalayensis* bites and further pre-clinical testing of antivenoms is needed [32].

*Naja mandalayensis* is a spitting cobra. A traditional remedy for venom ophthalmia involves using the leaf juice of tamarind (*Tamarindus indica*), chewed by the affected person or bystanders and dripped into the envenomed eyes [18], though this practice should be discouraged in favour of the standard snakebite treatment and management procedures endorsed by the World Health Organization [41]. Slowinski [43] described being spat at in both eyes by *N. mandalayensis*, resulting in immediate intense burning, blurred vision, and conjunctivitis. Despite irrigation with water and application of tamarind leaf juice, pain persisted for several hours, though full recovery and return of vision occurred the same day. Ocular envenomation by *N. mandalayensis* is commonly associated with acute symptoms such as eye pain, redness, swelling, and itching, typically resolving overnight [32]. Eight cases of venom ophthalmia presented with severe burning pain, profuse lacrimation, conjunctivitis, itchiness, and palpebral edema [44]. Symptoms resolved within three days in even the most severe cases, with no reports of lasting visual impairment.

### Recently confirmed occurrence

#### *Naja fuxi* Shi, Vogel, Chen, & Ding, 2022 – Brown banded cobra.

**Fig 4:**
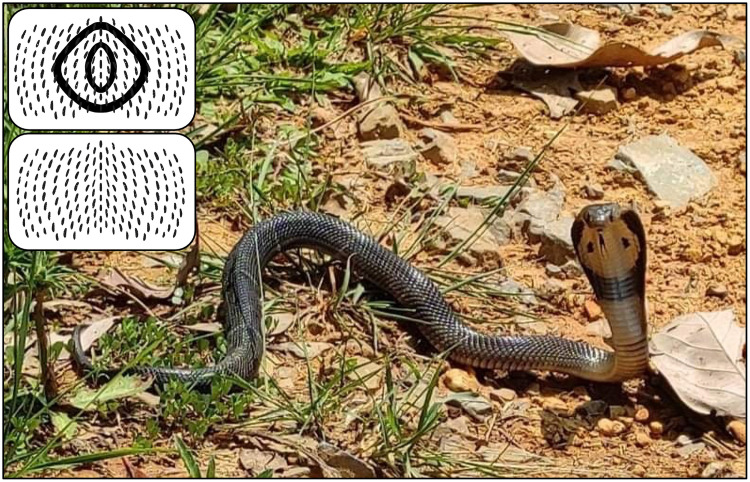
*Naja fuxi* photographed in Mandalay Region, Myanmar in April 2022 by K. **F.** **Wang (**https://www.inaturalist.org/observations/145051800, S1 Appendix**, record #2), modified by authors, used with permission under CC BY 4.0.** Hood marks may be O-shaped or absent (illustrated, top left), and the photographed individual shows brown body coloration with light bands on the posterior body, typical of this species.

Distribution – China (Yunnan and Sichuan provinces), Thailand (northern and northeastern), Vietnam, Laos, Cambodia. In Myanmar, *N. fuxi* is currently known from one record in Mandalay Division (Fig 4) and five records in Shan State (S1 Appendix). Two records immediately adjacent to the Myanmar border in Yunnan Province, China, suggest the presence of *N. fuxi* in Kachin State. *Naja fuxi* is likely distributed throughout many high elevation regions in Myanmar, and it is known to occur throughout the mountainous regions along the Myanmar-Thailand border in adjacent Thailand [21].

Description – Maximum reported adult snout-vent length of 1541.6 mm [21]. Dull scalation, with individual and ontogenetic variation in patterns. Juveniles are dark in body coloration with conspicuous white bands starting at the mid body and terminating at the tail tip. Bands may be present in some adults in Indochina, but generally fade away in many individuals giving the appearance of a dark unicolored snake in adulthood [21]. Some geographic variation in phenotype may be present, with adults from China generally being lighter in overall coloration and frequently displaying banding in adulthood [20]. Hood mark is an O-shaped white monocle bordered both inside and out by black coloration, and is often faint and may be absent altogether [21].

Natural History – Most activity occurs during the day [21]. Occurs in mountainous areas, dry evergreen forest, deciduous dipterocarp forest, forest edge habitats, and associated human settlements [20,21], but may also occur in atypical areas unexpected for the species such beaches and coastal habitats on islands or the mainland. Known to feed on frogs, snakes, birds, and rodents, and has been reported to enter villages to eat domestic fowl [20]. Confirmed prey species in Thailand include a spotted slug snake (*Pareas macularius*) and a water skink (*Tropidophorus thai*), excised from road-killed cobras [21]. Dispersing juveniles are most frequently observed in August and September in north and west Thailand, but between April and July in Nakhon Ratchasima, Thailand, suggesting that reproductive timing may show variation across the distribution [21].

Medical Importance – Due to its recent description, *Naja fuxi* has not yet been assessed by the World Health Organization [31], but it is anticipated to qualify as a Category 1 or Category 2 medically important snake throughout its distribution. Prior to its description, *Naja fuxi* was considered part of *N. kaouthia*, which is classified as a Category 1 medically important species in Myanmar [31]. No confirmed medical data attributable to *N. fuxi* currently exist from Myanmar, however, the species is presumed to be of considerable medical concern where it occurs. In an analysis of 126 snakebite cases from Xishuangbanna, Yunnan Province, China (2007–2014), *N. fuxi* was implicated in at least 11.9% (n = 15) of bites, making it the most frequently encountered species in the study region [45].

*Naja fuxi* is considered to be a non-spitting cobra. Shi et al. [20] reported that the species lacks specialized fang morphology for venom spitting, though they documented a single instance of venom being expelled at approximately 0.6 meters. Similarly, Ratnarathorn et al. [21] describe *N. fuxi* as non-spitting but note that some individuals may eject droplets of venom. No published reports exist for venom ophthalmia caused by *N. fuxi*, but clinical presentation and treatment are expected to be consistent with other cases of ocular *Naja* envenomation.

#### *Naja siamensis* Laurenti, 1768 – Indochinese spitting cobra.

**Fig 5:**
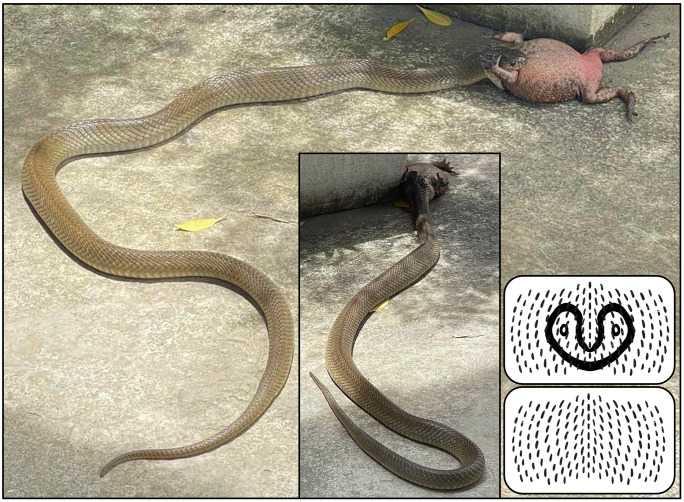
*Naja siamensis* feeding on *Duttaphrynus melanostictus* photographed in Tachileik, Shan State, Myanmar in December 2022 by Moeyan Paing, used with permission under CC BY 4.0 (S1 Appendix, record #213; main photo and inset photo). Hood marks may be U-, V-, or spectacle shaped or absent (illustrated, bottom right), and the photographed individual shows a patternless olive or light brown body typical of adults of this species.

Distribution – Thailand (central, northern, northeastern, and eastern), Cambodia, Laos, Vietnam. *Naja siamensis* has long been expected to occur in Myanmar [19], though specimens or credible observations of this species have remained lacking. A report of a spectacled cobra by Wall & Evans [46] from Myaungmya, Irrawaddy State, Lower Burma may be attributable to *N. siamensis*, as a spectacle-shaped hood mark would be atypical for the expected *N. kaouthia*, though this location is a great distance from existing *N. siamensis* records. Slowinski [43] mentioned reports from experienced snake collectors of a second species of spitting cobra in the country possessing a spectacle-shaped hood mark, almost certainly attributable to *N. siamensis*. In Myanmar, *N. siamensis* is currently known from three records in Kayin and Shan states (Fig 5), and the species may have a broad distribution along the Thailand border.

Description – Maximum reported adult total length of 1600 mm [47] though most adults measure between 900–1300 mm [19]. *Naja siamensis* exhibits an exceptional diversity of color morphs, with considerable geographic structuring associated with their occurrence [19]. In much of the distribution (including Burmese specimens), *N. siamensis* has a low contrast pattern with snakes generally being uniformly light brown or olive, with a conspicuous U-shaped, V-shaped, or spectacle shaped hood mark. In many areas on the Central Plain of Thailand, *N. siamensis* possesses a brightly contrasting black and white pattern with much variation in shape (U-, V-, H-, or spectacle shaped) and presence of hood mark. In southeastern and western Thailand, this species may be entirely black, with or without the spectacle shaped hood mark, and appear glossier than conspecifics from elsewhere.

Natural History – Habitat includes deciduous and evergreen forests, and agricultural areas, often in association with termite mounts [26]. This species is typically found in dryer and more upland areas than the co-occurring *N. kaouthia* [19]. This species is known to feed on snakes, frogs, small mammals, and birds [26], and known prey items in Myanmar include the Asian common toad (*Duttaphrynus melanostictus*; Fig 5). In Thailand, breeding occurs from March to July with clutches of 5–28 eggs being laid from May to September and hatching from July to November following a 55–80 day incubation period [26]. Neonates weigh 4.0–13.6 g and measure 200–320 mm in length upon hatching [30].

Medical Importance – *Naja siamensis* is classified as a Category 2 medically important snake in Myanmar [31]. While no clinical data specific to Myanmar are currently available, the species is presumed to be of considerable medical relevance where it occurs. In a national hospital-based survey conducted in Thailand during the 1980s, 114 (10%) of 1,145 dead snakes brought to hospitals with bite victims were identified as *N. siamensis* (then referred to as ‘*N. atra*’), compared to 83 (7.2%) identified as *N. kaouthia* [48]. Neurotoxic symptoms such as ptosis and respiratory distress occurred in 10.5% of *N. siamensis* cases, slightly higher than the 8.4% observed for *N. kaouthia*. Localized swelling and necrosis were common following bites from both species. One case report from Thailand described a 13-year-old girl bitten by *N. siamensis*, who experienced immediate pain and swelling but no neurological symptoms, with tissue necrosis developing at the site of the bite [19].

*Naja siamensis* is a spitting cobra. A case of venom ophthalmia in Thailand involved a 29-year-old woman who was sprayed in both eyes from approximately one meter away. Despite immediate irrigation with water, she experienced sharp pain, stinging, and irritation. One-hour post-exposure, she showed conjunctival injection, bilateral epiphora, leucorrhea, and swelling of the left eyelid, although her visual acuity remained normal [19]. Another case from Vientiane Province, Laos, described a 45-year-old man sprayed in the eyes from two meters away. He immediately immersed his head in a flowing river and sought medical attention two hours later. On examination, he had persistent bilateral eye pain and conjunctival injection but no corneal damage. He was treated with topical epinephrine and fully recovered within 24 hours [40].

#### *Naja sumatrana* Müller, 1997 – Equatorial spitting cobra.

**Fig 6:**
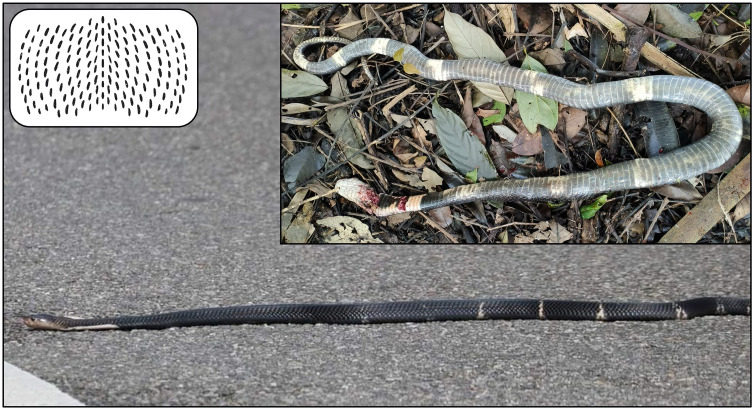
*Naja sumatrana* photographed in Ranong Province, Thailand (ca. 6 km east of the Kra Buri River, demarcating the Myanmar-Thailand border) on 7 November 2021 by Niran Anurakpongsathorn (https://www.inaturalist.org/observations/130079732, S1 Appendix, record #214), modified by authors, used with permission under CC BY 4.0. Inset photo shows specimen of *N. sumatrana* (S1 Appendix, record #215) photographed on 23 July 2022 at Lenya National Park, Tanintharyi Division, Myanmar by Htoo Nay Aung, used with permission under CC BY 4.0. Hood marks are absent (illustrated, top left), and both photographed individuals show black body coloration with widely spaced light bands, typical of adults of this species.

Distribution – Thailand (Malay Peninsula), Malaysia (Peninsular Malaysia, Sarawak, and Sabah), Singapore, Indonesia (Kalimantan, Sumatra), Brunei, Philippines (Palawan). In Myanmar, *N. sumatrana* is currently known from a single record in Lenya National Park (Fig 6), and the species is expected to be distributed throughout evergreen forest habitats in southern Tanintharyi Division.

Description – Maximum reported adult total length of 1480 mm [26]. Two color morphs of this species are present in adjacent Thailand i) a yellowish (golden) to light brown form and ii) a black color form, with neither form possessing a hood mark [26].

Natural History – This snake is nocturnal and crepuscular in habits. Habitat includes evergreen forests, agricultural areas, and anthropogenic developments [26,49]. Known to consume amphibians, reptiles, small mammals, and birds [26], and confirmed prey items include dog-toothed cat snake (*Boiga cynodon* [50]) and freshwater eel (*Anguilla borneensis* [51]). In Thailand, breeding occurs from November to March, with clutches of 7–10 eggs laid from December–April and hatching from February–June after a 69–73 day incubation period [26]. Neonates weigh 20–21 g and measure 340–370 mm in length upon hatching [30].

Medical Importance – *Naja sumatrana* is classified as a Category 2 medically important snake in Myanmar [6,31]. Thai *Naja kaouthia* monovalent antivenom has demonstrated immunoreactivity against *N. sumatrana* venom [52], suggesting potential cross-neutralization. Accordingly, BPI Cobra Antivenom may offer some level of efficacy, although this remains untested. In contrast, Thai Neuro Polyvalent Snake Antivenom (raised against *Bungarus candidus*, *B*. *fasciatus*, *N. kaouthia*, and *Ophiophagus hannah*) showed moderate neutralization activity against *N*. *sumatrana* phospholipase A₂ (PLA₂) and long-chain neurotoxins, but weak activity against short neurotoxins and cardiotoxins [53].

*Naja sumatrana* is a spitting cobra. Three documented cases of venom ophthalmia from Singapore presented with corneal punctate epithelial erosions, conjunctival injection, and chemosis. All individuals recovered fully within one week following immediate irrigation and clinical management with topical antibiotic prophylaxis and lubricants [54]. Two additional cases from Singapore reported the use of topical heparin as an adjunct treatment when traditional irrigation failed to provide symptom relief [55]. In the first case, a 57-year-old male was sprayed in both eyes, resulting in blurred vision, throbbing pain, conjunctival injection, and blepharospasm. After limited improvement from bilateral irrigation with normal saline, 1 mL of unfractionated heparin (5000 IU/mL) was administered to both eyes, leading to marked symptom relief. In the second case, a 21-year-old male experienced pain and tearing after being sprayed in the right eye. Following initial self-irrigation, he received a total of 9.5 L of normal saline irrigation in hospital, after which 1 mL of unfractionated heparin (5000 IU/mL) was instilled. He was discharged with topical antibiotics and lubricants and recovered fully.

### Possible occurrence (unconfirmed)

#### *Naja sagittifera* Wall, 1913 – Andaman cobra.

**Fig 7:**
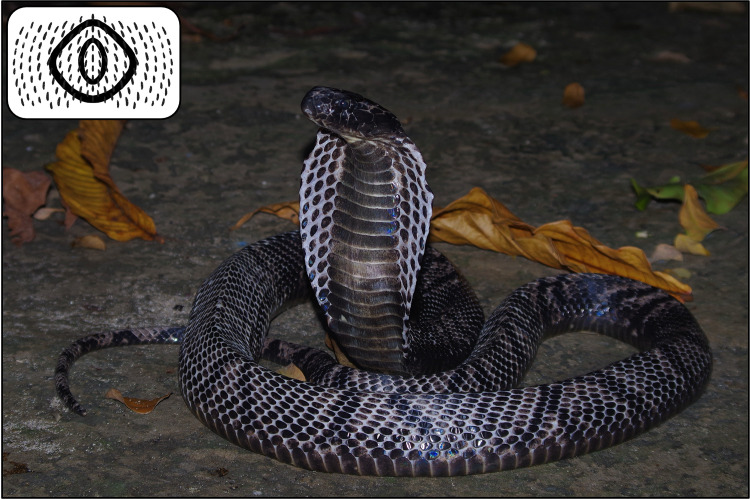
*Naja sagittifera* photographed in Swaraj Dweep (Havelock Island), Andaman Islands, India in January 2023 by Yatin Kalki, used with permission under CC BY 4.0. Hood marks are O-shaped (illustrated, top left), and the photographed individual shows grey to black body coloration with dark crossbanding, typical of adults of this species.

Distribution – Andaman and Nicobar Islands, India; known from North, Middle, and South Andaman Island ([25], S. R. Chandramouli, pers. com.; Fig 7). *Naja sagittifera* is not known to occur in Myanmar, though it may be present in the Coco Islands. The endemic Anderson’s pitviper (*Trimeresurus andersoni*), previously known only from the Andaman Islands and Car Nicobar, India has recently been documented from Great Coco Island, Myanmar [9]. Consequently, it is plausible that other Andaman snake fauna may share similar biogeographic distributions to *T. andersoni* [56], and continued search efforts may produce evidence of *N. sagittifera* in the Coco Islands, Myanmar.

Description – Reported adult total length >1500 mm [25]. Scales are smooth and matte, and patterns are variable. Adults may be i) entirely black above with hood scales edged occasionally with pale grey, ii) pale slate-grey with narrow, irregular white, or ash-grey crossbanding (may or may not be forked on sides of body), head black or dark grey, and dorsal surface of hood with eye-shaped hood mark, or iii) light cream with irregular black or grey patches, pale grey underside with scales sometimes powdered with darker grey, throat or ventral side of neck may have a broad and dark cross band. A distinct monocle shaped hood mark is present on the hood [24]. Adults have less distinct markings than juveniles [25], which are dark black in coloration with 34–40 white cross-bars [24].

Natural History – Believed to be largely diurnal. Habitat includes thick forest, mangrove forest, forest edges [25], and areas of human habitation [24]. Adults of this species are believed to feed primarily on rodents and have been recorded taking domestic duck and chicken eggs, while juveniles are believed to consume amphibians and lizards. The alimentary canal of a dissected juvenile yielded insects, suggesting it had likely consumed and digested a frog [57], and another juvenile was observed feeding on road-killed frogs at night in October [24]. Egg laying occurs in May, with nesting occurring in rat holes and termite mounds where the female probably remains with the clutch over the incubation period [25].

Medical Importance – *Naja sagittifera* is classified as a Category 2 medically important snake in India, but is not listed for Myanmar [31]. Antivenoms specifically produced for this species are unavailable. Preclinical studies have demonstrated poor cross-neutralization by Indian antivenom (Bharat Serums and Vaccines Ltd.; raised against *N. naja*) and Thai antivenom (Queen Saovabha Memorial Institute; raised against *N. kaouthia*), suggesting that currently available products may be inadequate for treating envenomation by this species [58]. No cases of human envenomation have been documented, and snakebites attributed to *N. sagittifera* remain unknown [25].

*Naja sagittifera* is considered to be a non-spitting cobra, though behavioral data are lacking for this species.

### Human-cobra encounters in Myanmar

**Fig 8:**
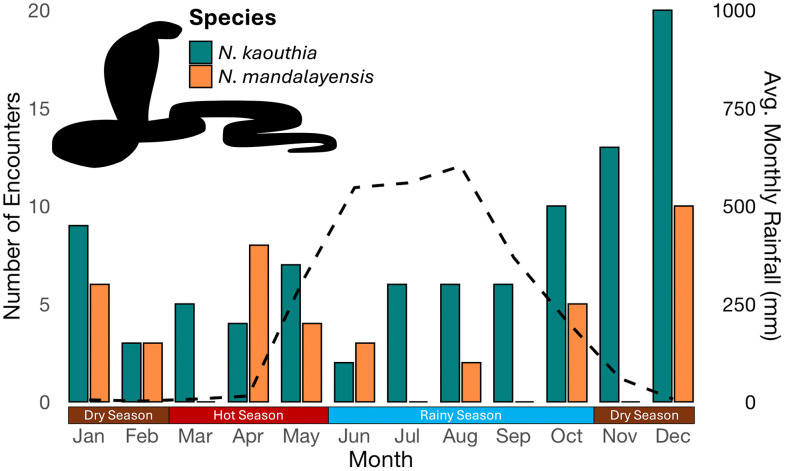
Distribution of reported encounters with *Naja kaouthia* (n = 91) and *Naja mandalayensis* (n = 41) across the year throughout Myanmar with average monthly rainfall for Yangon shown. Note that most reported encounters occur during the dry season.

Cobras are major contributors to snakebite envenomation in Myanmar, with *Naja kaouthia* and *N. mandalayensis* classified as Category 1, and *N. sumatrana* and *N. siamensis* as Category 2 medically important species [31]. Due to their synanthropic tendencies and tolerance for disturbed habitats, cobras thrive in proximity to humans, increasing the likelihood of human–cobra encounters compared to less disturbance-tolerant species [59]. Our encounter data (Fig 8) indicate non-random temporal structure in reported cobra observations. Monthly encounter frequencies deviated significantly from a uniform distribution for *Naja kaouthia* (χ²(11, N = 91) = 35.73, p < 0.001), with reported encounters highest in December (n = 20) and November (n = 13), and lowest in June (n = 2) and February (n = 3). *Naja mandalayensis* also exhibited significant monthly variation (χ²(11, N = 41) = 28.41, p = 0.003). Reported encounters for this species were highest in December (n = 10) and April (n = 8), with no records in March, July, September, or November.

When months were grouped into seasons and expected frequencies were adjusted to account for unequal seasonal duration, seasonal variation remained significant for *N. kaouthia* (χ²(2, N = 91) = 10.64, p = 0.005), with reported encounters elevated during the dry season (n = 45) relative to expectation. In contrast, seasonal variation was not statistically significant for *N. mandalayensis* (χ²(2, N = 41) = 4.89, p = 0.087), although dry-season encounters were numerically higher (n = 19). A chi-square test of independence detected no significant difference in seasonal distribution between species (χ²(2) = 2.55, p = 0.28), suggesting broadly similar seasonal encounter likelihood across the year.

However, these records derive from opportunistic citizen science submissions and therefore reflect reported encounters rather than standardized measures of snake activity. While the significant deviations from uniformity indicate structured temporal reporting patterns, they do not allow discrimination between biological seasonality and seasonal shifts in detection probability, observer effort, or human behavior. Reported encounter patterns differ from capture records of *N. kaouthia* in Thailand, where 57% of individuals were recorded during the wet season months (May–October) [28]. As both datasets are influenced by human-mediated detection and differ methodologically, this contrast should not be interpreted as evidence of opposing biological seasonality. Rather, it underscores that temporal patterns may vary depending on sampling framework and reporting context.

Although metadata were often limited, many observations appeared to occur in or near human habitation based on visible environmental context. However, this likely reflects observer distribution and reporting bias, as most contributors are concentrated in residential settings. Consequently, the predominance of residential encounters cannot be interpreted as evidence that cobras preferentially occupy anthropogenic environments during different seasons. While it is plausible that seasonal environmental pressures, including shifts in prey availability, could influence cobra movements between natural and anthropogenic habitats, our dataset does not permit this hypothesis to be tested directly. Structured ecological surveys incorporating standardized sampling effort and detection correction would be required to disentangle biological activity patterns from reporting bias.

Finally, the seasonal distribution of snakebite incidence likely differs from the encounter patterns presented here. Previous surveys in Mandalay Division [60] indicate that most snakebites occur during agricultural or forest-related activities. As these occupational exposures are themselves seasonal, snakebite incidence likely reflects patterns of human labor and exposure risk rather than residential encounter frequency. Moreover, many snakebite cases may not be represented in the citizen science platforms used in this study. Therefore, encounter rates derived from our dataset should not be interpreted as predictors of snakebite seasonality.

### Barriers to effective snakebite treatment in Myanmar

Multiple barriers complicate the treatment of venomous snakebite in Myanmar, impacting envenomation cases from cobras as well as other medically important species. One of the most direct challenges is antivenom efficacy and availability. Currently, only two antivenoms are produced and distributed by Burmese Pharmaceutical Industries (Myanmar Pharmaceutical Factory), the sole domestic producer [10]. These include monovalent products raised against Siamese Russell’s viper (*Daboia siamensis*) and monocled cobra (*Naja kaouthia*), offering limited cross-neutralization across the broader diversity of venomous snake species present in the country. Studies have demonstrated that even among Asian cobra species, antivenom efficacy is often reduced when used against allospecifics [11] or geographically distant populations of the same species [13,34]. These findings highlight the need for regionally tailored antivenoms to effectively treat snakebite cases in Myanmar. In addition to efficacy, challenges in antivenom availability and distribution further hinder treatment outcomes. Barriers such as logistical constraints, healthcare infrastructure limitations, and rural access issues can considerably delay or prevent the administration of antivenom, especially in countries with limited resources [61–63].

Community-level knowledge and perceptions of snakebite also play a substantial role in bite outcomes. A study from the Mandalay region [60] reported inadequate awareness surrounding snakebite symptoms and treatment across surveyed communities. While most respondents could identify dangerously venomous snakes and noted that bites often occurred during agricultural or forest-related activities, knowledge about first aid and symptom recognition was lacking. Only 39% of surveyed individuals were aware of correct first aid procedures, while over 60% recommended using a tourniquet, a harmful method that can worsen envenoming outcomes. Encouragingly, 88% of respondents said they would take a bite victim to a government hospital, with 58% citing antivenom availability as their primary motivation in doing so. However, many individuals also acknowledged traditional treatment methods, and 25% mentioned at least one harmful practice they would consider using.

Traditional snakebite remedies vary in their level of harm, but range from being ineffective to dangerous [64]. Harmful practices reported in Madaya and Kyaukse townships included attempting to suck out venom, cutting or tattooing around the bite site, burning the wound, drinking substances to induce vomiting, or massaging traditional medicine into the bite area [60]. Less harmful (lower likelihood of additional infection or trauma) but still ineffective methods included applying a “black stone” to the wound, drinking holy water or coconut water, reciting prayers, or placing a dead chick on the bite site. A unique traditional remedy used for spitting cobras involves chewing leaves of the tamarind tree (*Tamarindus indica*) and dripping the juice into the eyes [18], allegedly inducing more pain than the venom itself [43]. These traditional practices often delay access to effective medical care, increasing the time for progression of venom symptoms or introducing secondary infection, and should therefore be strongly discouraged. The administration of antivenom in a medical facility remains the only efficacious treatment for individuals exhibiting venom-induced toxic effects [10,41,65–67]

### Medical management of cobra envenoming

Medical management of snakebite victims following envenomation from cobra species requires timely assessment of the envenomed victim and an understanding of available strategies required to mitigate venom-induced effects. Accurate species identification of the responsible snake, the circumstances of exposure, the victim’s previous medical history, use of appropriate first aid, and the availability and length of travel time to adequate medical resources are key factors affecting the medical outcome of the envenomed victim [41,60]. The systemic and local complications following envenomation by the *Naja* species presented here (*N. fuxi, N. mandalayensis, N. sagittifera, N. siamensis, N. sumatrana, N. kaouthia*) are primarily observed as neurotoxicity and cytotoxicity [41,68,69]. The monocled cobra (*Naja kaouthia*) has historically been the most recognized medically important species [33]. The main treatments required for favorable outcomes are antivenom and the ability to provide complete respiratory support.

### Medical background

Cobra bites account for a majority of envenoming to victims living in remote localities of Southeast Asia where travel times to adequate medical care are often lengthy [33,70]. Consequently, traditional methods involving religious beliefs, physical measures such as cutting into the bite wound, application of herbal remedies and use of medicinal plant extracts, or snake stones, are the initial forms of therapy used in rural communities [71–73]. Medicinal plant roots, leaves, rhizomes, seeds, and bark have been demonstrated to possess bioactive components derived from flavones, glycoproteins, and tannins that have pharmacological actions capable of neutralizing some toxic venom components of several cobra species (*Naja kaouthia, N. naja, N. nigricollis, N. sputatrix*), with actions similar to the neutralizing capabilities of antivenom [71]. Importantly, the initial use of these methods results in delayed time to appropriate medical care. To date, these traditional therapeutic strategies have no proven medical efficacy, and in some instances may worsen venom-induced effects. Research on the use of medicinal plant extracts is an ongoing process that may yield useful treatments for localized venom-induced tissue injuries [67,71]. The use of any of these treatment methods to resolve venom-induced pathophysiological manifestations should not preclude the pursuit of conventional healthcare [33].

### Pre-hospital support

First aid or pre-medical facility support following a cobra bite should be limited with the most important actions being to make sure the victim has no evidence of airway obstruction and transport the victim to adequate medical care as rapidly as possible. A compromised airway can be improved by placing the victim in the recovery position via head tilting to the side and making sure the tongue and fluids are not obstructing the passage of air. The use of tourniquets, herbal preparations, cutting, etc. are not recommended. For victims of confirmed cobra envenoming the immobilization of the bitten extremity, minimization of physical activity, and the appropriate application of pressure-bandage immobilization or pressure-pad bandage may be useful in retarding venom-induced effects during transport to medical care [10,33,41,65,73]. However, for these bandaging measures to be effective requires appropriate application of the bandage, which is often applied incorrectly [74]. In situations where the victim’s respiratory capacity is declining it is important to position the victim in the recovery position with head tilted so that salivary fluids or any solids do not block the upper airway [41]. If respiratory status is rapidly declining a bag-valve-mask may be applied to maintain ventilation [41,75,76].

### Medical management at the hospital

Conventional healthcare of cobra envenomed victims in a medical facility setting should involve the administration of antivenom (purified venom-antigen-binding immunoglobulins derived primarily from the plasma of equines that have been hyperimmunized with snake venom), which is the specific medical pharmacotherapy for treating victims exhibiting venom-induced toxic effects [10,41,65–67]. Ideally, the appropriate antivenom used should contain antibodies directed against key toxins present in the venom of the responsible cobra species if the patient is to have a confirmed positive therapeutic response [67,77]. Monovalent and polyvalent antivenoms for medical management of victims envenomed by various species of Asian and Southeast Asian *Naja* spp. are available, with para-specific coverage provided by several different antivenom products [66,68,69,78]. Importantly, for antivenom to be optimally effective, accurate identification of the snake responsible for envenoming is required (when possible), which should dictate the selection of the appropriate antivenom. In situations where antivenom is unavailable intense supportive care in the form of respiratory and renal support, and cardiovascular resuscitation may need to be implemented, and fluid volume repletion measures required to maintain homeostasis [41,65,76].

### Antivenom

Administration of the appropriate antivenom (or antivenom with para-specific neutralizing antibodies) should be initiated intravenously as soon as possible in a hospital setting following envenoming. Timely antivenom administration can halt the development or progression of cobra venom-induced neurotoxicity as evidenced by symptoms such as ptosis, aphasia and dysphagia, which can progress to bulbar paralysis or respiratory failure [41,69,79]. If the victim presents with restrictive pressure immobilization bandaging or pressure pad bandaging it should be left in place until antivenom administration has been initiated [23,41,65,73,80]. Antivenom should be appropriately diluted and slowly administered initially to minimize potential acute allergic reactions in addition to close patient observation. Acute reactions are typically not IgE mediated but due to the activation of complement, which is induced by the occurrence of antibody and protein aggregates, or rapid intravenous administration of a large antivenom dose [81]. Prophylactic use of epinephrine prior to antivenom administration has been reported to reduce early severe allergic complications [41,82]. Epinephrine given intramuscularly or intravenously may also be required when allergic reactions occur post antivenom administration [41]. Antivenom effectiveness depends on the venom dose injected, antibodies that recognize venom toxins responsible for toxic effects clinically, and the antivenom dose [77]. Although antivenom manufacturer product information sheets may provide dosing guidelines they are not always based on data derived from controlled clinical trials and laboratory animal data [67,77,83]. As such, antivenom dosing may be to some extent empirical, with dosing titrated to patient response (reduced neurotoxicity or halting of coagulopathy) [41,69]. Antivenoms produced using the venom of a single snake species (monovalent or monospecific) or several snake species (polyvalent or polyspecific) in immunization protocols provide an array of neutralizing antibodies against venom antigens [67,83]. Antivenoms with indications discussed here have only an indication for a single species, *Naja kaouthia*: QSMI Cobra and Neuro Polyvalent Snake Antivenin (Queen Saovabha Memorial Institute, Bangkok, Thailand) and anti-NK Antivenom (Myanmar Pharmaceutical Factory - MPF; formerly Burma Pharmaceutical Industry - BPI) [41,84]. South African Institute for Medical Research Polyvalent antivenom has been found to be ineffective for *N. kaouthia* envenoming [85]. Species-specific antivenom is not available for *N. fuxi, N. mandalayensis*, *N. sagittifera*, *N. siamensis*, or *N. sumatrana*.

Cross-neutralization properties of some antivenoms with the venoms of different cobra species have been reported, which may provide some insight to potential beneficial antibody-venom toxin binding effects [14,32,52,66]. Importantly, pre-clinical cross-neutralization studies, whether *in vitro* or *in vivo*, may not translate to clinical effectiveness. Confounding components with the use of antivenoms in treating patients envenomed by non-indicated cobra species are related to geographic venom variability within a single cobra species, variation in manufacturer antivenom batch production, and the health history of the envenomed patient, all of which can further compromise the potential para-specific efficacy of a given antivenom. Unfortunately, the reporting of cases with antivenom use and medical details of envenoming to humans by *N. fuxi* and *N. sagittifera* are not available, and are quite limited for *N. mandalayensis*, *N. siamensis*, and *N. sumatrana*. As such the interpretation of laboratory studies and the use of non-specific antivenom should be carefully considered. Close patient observation with no development or evidence of symptoms indicates antivenom administration should be withheld.

Anti-NK antivenom (MPF/BPI) has been demonstrated to effectively neutralize *N. kaouthia* and *N. mandalayensis* venom-induced effects in murine lethality studies [14]. Clinically, an *N. mandalayensis* envenomed patient with persistent neurotoxicity was treated with anti-NK antivenom (8 vials) and was reported to have had a positive therapeutic response [32]. During the Myanmar Snakebite Project, BPI Cobra antivenom and “Indian-sourced antivenoms” were used in the treatment of envenomed victims; however, details with respect to which antivenoms were used and effectiveness in either cases of *N. kaouthia* or *N. mandalayensis* envenoming were not provided [10]. The QSMI Cobra Monovalent antivenom has been demonstrated in murine lethality experiments to possess varying degrees of cross-neutralizing actions against the venom-induced lethal effects of venoms from *N. sumatrana* from several different geographic regions [52]. A large retrospective cohort study that included cases of *N. siamensis* envenoming suggests possible therapeutic effects following the administration of QSMI antivenom may potentially reduce fatalities [68]. Additional reported cases of *N. siamensis* envenoming with neurotoxicity were treated with QSMI monovalent cobra or QSMI neuro polyvalent antivenom, but recurrent neurotoxicity developed, and repeated antivenom dosing was required due to prolonged symptoms [37,78]. A review of multiple pre-clinical laboratory studies reported antivenom-venom cross-neutralization of Haffkine, Bharat, and VINS antivenom with *N. kaouthia*, QSMI antivenom with *N. siamensis*, and Bharat, VINS, QMSI, and SABU (Serum Anti Bisa Ular - Biosave, Indonesia) with *N. sumatrana* [66]. *Naja kaouthia* envenoming to a 35-year-old male with venom-induced neurotoxicity was successfully treated with 25 vials of VINS Snake Venom Antiserum I.P. (Asia) [37]. Collectively, the above pre-clinical studies and case reports involved the testing or use of antivenoms for non-indicated cobra species and provide a limited perspective and the use of antivenom that has involved envenoming from a non-indicated species should be carefully weighed with respect to patient benefit versus risk.

### Respiratory management

Respiratory complications from venom-induced neurotoxicity can progress to respiratory failure due to respiratory muscle weakness and total muscle paralysis, requiring complete respiratory support via assisted mechanical ventilation [68,78,79,85]. Intubation coupled with mechanical ventilation is not uncommon in cases of Southeast Asia cobra (*Naja* spp.) envenoming and is necessary to sustain the life of envenomed victims [41,68,69,75]. These measures are often implemented prior to, or concurrently with, antivenom therapy [79]. However, when antivenom is not readily available respiratory support alone can sustain life [41,75]. In facilities where mechanical ventilatory support is not available the intubated patient can be maintained with the manual use of an Ambu bag or bag-mask or bag-mask-valve ventilation [41,75]. Recurrent respiratory failure several hours after antivenom treatment and removal of respiratory support has been described in cases of *N. kaouthia* and *N. siamensis* envenoming, which required reintubation and further antivenom dosing [78,86]. Given the descending paralysis from neurotoxicity that progresses to loss of respiratory function the use of acetylcholinesterase inhibitor drugs can provide respiratory improvement of paralytic symptoms and maintenance of respiratory function. Acetylcholinesterase inhibitors such as neostigmine allow for acetylcholine to be available and active at the neuromuscular junction and halting the paralysis of respiratory muscles [41,65,79,85]. When respiratory compromise is observed initial administration of intravenous atropine and continuing with follow-up neostigmine given intramuscularly is recommended. Given the relative short half-life (≈ 30 mins) of neostigmine repeat dosing may be required to maintain adequate victim ventilation [41,87].

### Bite wound care

Local wound tissue damage and necrosis can result from venom cytotoxin-induced effects and represent a serious clinical concern following Asian cobra envenoming, which can be further compounded by bacteriological infections [69,88,89]. *Naja kaouthia*, *N. mandalayensis*, *N. siamensis*, and *N. sumatrana* all have documented envenoming cases of clinical cytotoxicity as evidenced by the development of considerable tissue necrosis, and it is presumed *N. fuxi* and *N. sagittifera* envenoming may be capable of causing similar complications [32,69,88,89]. Positive bacterial cultures are common and have been reported to have involved 48 different bacterial strains [88]. As such wound care should be guided by bacteria culture and follow-up treatment with antibiotics [69,85]. Ciprofloxacin and amoxicillin/clavulanate have been used in *N. kaouthia* cases of secondary infection involving *Morganella morganii* and *Enterococcus faecalis,* and aminoglycosides, 3rd and 4th generation cephalosporins, fluroquinolones, and doxycycline have also been used [69,76,85,88]. Additionally, tetanus toxoid should be administered [41,76]. In severe cases when antivenom treatment is delayed or infection is confirmed with necrotic tissue surgical debridement of necrosed tissue may be necessary [41,68,69,76]. Invasive surgery (fasciotomy) due to concerns of compartmental hypertension (compartment syndrome), although frequently performed in Asian and Southeast Asian *Naja* spp. envenoming cases is not a recommended practice. Minimal delay to good medical support and proper administration of appropriate antivenom will usually mitigate the development of elevated intracompartmental pressure [41,65,68,69,76].

### Ocular exposures

*Naja mandalayensis* [32] and *N. kaouthia* [39] are recognized species with documented ocular exposures due to the spitting of venom. Two additional spitting cobra species ranging in Myanmar are *N. siamensis* [68,90], and *N. sumatrana* [54], and venom spitting behavior is also known from *N. fuxi* [20,21]. Venom-induced ophthalmia following ocular contact from venom ejected from the fangs (commonly referred to as spitting) is usually a transient medical complication involving excessive lacrimation, intense pain, blepharospasm, conjunctival discharge, eyelid swelling, itching, uveitis, and photophobia [32,39,91]. Most victims recover without permanent sequalae [41,68,91]. Immediate irrigation of the affected eye(s) with copious amounts of sanitary water or normal saline, and treatment with analgesic drugs such as acetaminophen/paracetamol should be instituted [39–41,90,91]. Allergic keratoconjunctivitis may also occur and the use of topical antihistamine is beneficial [40]. In rare instances corneal ulceration and secondary infection may appear, requiring the administration of antibiotic eye drops such as moxifloxacin [39,91]. The use of topical corticosteroids, or topical or intravenous antivenom, is contraindicated [40].

## Conclusions

The cobra fauna of Myanmar has long been a source of confusion, but ongoing research efforts and distributional data continue to bring clarity to species diversity in the country. We confirm the presence of five species in Myanmar based on voucher specimens and/or diagnostic photographic records: *Naja fuxi*, *N. kaouthia*, *N. mandalayensis*, *N. siamensis*, and *N. sumatrana*, and suggest the possible but unconfirmed presence of *N. sagittifera*. This extended species list underscore the underappreciated diversity of *Naja* in the country, emphasizes the need for continued survey work and biodiversity research, and highlights important implications for clinical management of snakebite envenoming. While most human-cobra interactions in Myanmar involve *N. kaouthia* and *N. mandalayensis*, the current antivenom product available uses only antibodies derived from the former. Concerns exist regarding the ability of the existing antivenom product to offer effective cross neutralization to treat bites from *Naja fuxi*, *N. mandalayensis*, *N. siamensis*, and *N. sumatrana*, and no experimental data exist at present to address this potential shortcoming. The presence of underappreciated cobra diversity in Myanmar complicates the therapeutic landscape, especially in the absence of species-specific antivenoms and broader preclinical antivenom testing, and regionally informed antivenom production is urgently needed. Reported human–cobra encounters in our dataset were most frequent during the dry season and commonly occurred near human settlements; however, because records derive from opportunistic citizen science submissions, these patterns likely reflect seasonal variation in human activity and detection probability as well as potential shifts in snake ecology. Regardless of the underlying drivers, the continued proximity of medically important cobras to human communities, combined with limited public awareness and the persistence of harmful traditional treatments, continues to impede timely and effective snakebite care.

Addressing the burden of cobra envenomation in Myanmar will require an integrated strategy combining continued scientific research of venomous snake and venom diversity, improved access to effective antivenom, and community-based education focused on first aid and prompt medical care. Continued scientific research and clinical surveillance are essential in informing both public health procedures and the development of appropriate therapeutics for snakebite in Myanmar, and we emphasize the need to advance efforts in both areas.

## Supporting information

S1 Appendix List of cobra observations used in this study. Abbreviations: CAS = California Academy of Sciences, MS = Myanmar Snakes Facebook group, NSCIM = Native Species Conservation and Identification in Myanmar Facebook group, UTADC = University of Texas at Arlington Amphibian and Reptile Diversity Research Center Digital Collection.
(XLSX)

S2 Appendix Burmese language abstract. (PDF)
