# Supplementary material for: The cobras (genus Naja) of Myanmar: An updated species list with information on identification, distributions, and medical importance
Source: PLoS Negl Trop Dis. 2026 Jun 29;20(6):e0014445. doi: 10.1371/journal.pntd.0014445 (PMC13340773; doi:10.1371/journal.pntd.0014445)
Supplement: S2 Appendix — (PDF) [file pntd.0014445.s002.pdf]

BURMESE LANGUAGE ABSTRACT: မြွေဟောက်မျိုးစုသည် မျိုးစုံမျိုးကွဲများပြားပြီး မျိုးစိတ်သစ်များလည်း ထွက်ပေါ်လျက်ရှိသော မျိုးစုဖြစ်ရာ မြန်မာနိုင်ငံရှိ မြွေဟောက်မျိုးများ၏ မျိုးစိတ်ကွဲပြားမှုသည်လည်း တိကျကောင်းမွန်သော သိပ္ပံစာတမ်းများ အရေအတွက်မြောက်များစွာထုတ်ဖော်ရေးသားနိုင်ခြင်း မရှိသောကြောင့် အချိန်ကာလရှည်ကြာစွာ တိကျသောအချက်အလက်များမရှိဘဲ ရှုပ်ထွေးနေခဲ့ရပါသည်။ ဒါ့အပြင် မြွေဟောက်များသည် ဆေးလောကအတွက်လည်း အလွန်အရေးကြီးသည့်မြွေအမျိုးအစားများဖြစ်သည့်အတွက် ဤကဲ့သို့ မသေချာမှု၊ ရှုပ်ထွေးမှုများသည် မြွေဟောက်ကိုက်ခံရမှုဆိုင်ရာ ပြည်သူ့ကျန်းမာရေး တုံ့ပြန်ဆောင်ရွက်မှုများအပေါ်တွင် ကြီးမားသောအဟန့်အတား ဖြစ်စေခဲ့ပါသည်။ ဤလေ့လာမှုစာတမ်းတွင် မြန်မာနိုင်ငံအတွင်း မတူညီသောမြွေဟောက်မျိုးကွဲများ၏ ပျံ့နှံ့တည်ရှိမှုဒေတာအချက်အလက်များ၊ သဘာဝသမိုင်းကြောင်းနှင့် ဆေးဘက်ဆိုင်ရာ သက်သေအထောက်အထားများကို ပေါင်းစပ်အသုံးချ၍ မြန်မာနိုင်ငံအတွင်းရှိ မတူညီသောမြွေဟောက်မျိုးကွဲများ၏ သိပ္ပံဆိုင်ရာအချက်အလက်များနှင့် ၎င်းတို့၏ ပျံ့နှံ့တည်ရှိမှုဆိုင်ရာစာရင်းအချက်အလက်များကို ရှင်းလင်းစွာဖော်ပြထားသည့်အပြင်၊ မြွေဟောက်အဆိပ်အကြောင်း၊ မြွေကိုက်မှုဆိုင်ရာ ကုသထိန်းချုပ်ပုံနှင့် အဆိပ်သင့်မှုဆိုင်ရာ နောက်ဆက်တွဲ ပြဿနာများကို တွက်ချက်၍ ရှင်းလင်းစွာ ထည့်သွင်းရေးသားထားပါသည်။ ထို့အပြင် ဤစာတမ်းတွင် မြန်မာနိုင်ငံ၌ မြွေဟောက်မျိုးကွဲ ၅ မျိုး (*Naja fuxi*, *N. kaouthia*, *N. mandalayensis*, *N. siamensis*, နှင့် *N. sumatrana*) ရှိကြောင်း အတည်ပြုနိုင်ခဲ့သည့်အပြင် ဇီဝမျိုးစုံမျိုးကွဲ ပြန့်နှံ့မှုဆိုင်ရာ အချက်အလက်နှင့် ဖြစ်တန်စွမ်းများကိုအခြေခံ၍ နောက်ထပ်မြွေဟောက်မျိုးစိတ်တစ်ခု (*N. sagittifera*) လည်း မြန်မာနိုင်ငံ၌တွေ့ရနိုင်ခြေရှိကြောင်း ဖော်ထုတ်ခဲ့ပါသည်။ စာတမ်းတွင်ကိုးကားထားသော မြွေနှင့်လူ ထိပ်တိုက်တွေ့ဆုံမှုမှတ်တမ်းများအရ တစ်နှစ်တာအတွင်း အချိန်ကာလအလိုက် သိသာထင်ရှားသော ကွဲပြားမှုများရှိနေပြီး၊ ဒီဇင်ဘာလနှင့် ခြောက်သွေ့သော ရာသီများတွင် ထိပ်တိုက်တွေ့ရှိမှုအများဆုံး ဖြစ်နေကြောင်း တွေ့ရပါသည်။ ယခုရရှိထားသော အချက်အလက်များသည် အခွင့်သင့်သလို ပေးပို့လာကြသည့် အများပြည်သူပူးပေါင်းပါဝင်မှုဆိုင်ရာ သိပ္ပံမှတ်တမ်းများအပေါ် အခြေခံထားခြင်းကြောင့်၊ ဤတွေ့ရှိမှုပုံစံများသည် မြွေများ၏ ဇီဝကမ္မဆိုင်ရာ ရာသီအလိုက်လှုပ်ရှားမှုများထက် လူတို့၏လှုပ်ရှားမှုနှင့် ရာသီအလိုက်ကွဲပြားသော မြွေတွေ့ရနိုင်ချေများအပေါ်သာလျှင်မူတည်နေကြောင်း ထင်ဟပ်စေနိုင်ပါသည်။ အခြားဒေသများတွင် မိုးရာသီ၌ မြွေတွေ့ရှိမှု အများဆုံးဖြစ်သည်ဟု ဖော်ပြချက်များနှင့် ကွဲလွဲနေခြင်းသည် ဒေတာကောက်ယူသည့် ပုံစံစနစ် (sampling framework) က ရလဒ်အဖြေများအပေါ် မည်မျှအသက်ရောက်မှုရှိသည်ကို မီးမောင်းထိုးပြနေခြင်းပင် ဖြစ်ပါသည်။

ဆေးလောကဆိုင်ရာအချက်အလက်များနှင့် မြွေဆိပ်ဆိုင်ရာအချက်အလက်များအရ လက်ရှိတွင် မြန်မာပြည်တွင်းဖြစ် မြွေဆိပ်ဖြေဆေးမှာ မြွေဟောက်တစ်မျိုးတည်းအတွက်သာ ကိုယ်စားပြုထားကြောင်းလေ့လာရပါသည်၊ သို့ဖြစ်ရာ မြွေဟောက်မျိုးကွဲ၅မျိုးရှိသော နိုင်ငံတစ်နိုင်ငံအနေဖြင့် အဆိုပါမြွေဆိပ်ဖြေဆေးသည် အခြားမျိုးစိတ်များအတွက်မှ အဆိပ်ဖြေနိုင်စွမ်း အကန့်အသတ်ရှိနိုင်သည့်အတွက် ထည့်သွင်းစဉ်းစားရန်လိုအပ်ပါသည်။ ထို့အပြင် မြန်မာနိုင်ငံနေရာအနှံ့အပြားတွင် ဆေးမြီးတိုကုသမှုများ ရှိနေဆဲဖြစ်ပြီး၊ အချို့သော ဆေးမြီးတိုကုသမှုများမှာ အဆိပ်ကိုပိုဆိုးစေသော အန္တရာယ်ရှိနည်းလမ်းများပါဝင်နေသည့်အတွက် ထိရောက်သော သိပ္ပံနည်းကျ ဆေးကုသမှုများကို ကြန့်ကြာစေကာ လူနာအခြေအနေကို ပိုဆိုးစေပါသည်။ ဆေးဘက်ဆိုင်ရာ သက်သေအထောက်အထားများအရ အာရုံကြောဆိုင်ရာ အဆိပ်သင့်ခြင်း၊ အသက်ရှူလမ်းကြောင်းဆိုင်ရာ အခက်အခဲဖြစ်ခြင်းနှင့် တစ်ရှူးများ ပျက်စီးရခြင်းတို့သည် မြွေဟောက်အဆိပ်သင့်ခြင်း၏ အဓိကနောက်ဆက်တွဲပြဿနာများဖြစ်ပြီး၊ အချို့သောပြင်းထန်သောအဆိပ်သင့်မှုများတွင် အဆိပ်ဖြေဆေးဆိုင်ရာကုထုံးများ၊ အသက်ရှူလမ်းကြောင်းဆိုင်ရာ အောက်ဆီဂျင်ပိုက်အကူအညီများ၊ ရောဂါပိုးကူးစက်မှုစီမံခန့်ခွဲမှုနှင့် ခွဲစိတ်ကုသမှုများပင် လိုအပ်ကြောင်း ပြသထားပါသည်။အထက်တွင်ပြောခဲ့သကဲ့သို့ မြန်မာနိုင်ငံရှိ မြွေဟောက်များ၏ ရှေးယခင်ကတရားဝင်အသိအမှတ်ပြုထားသည်ထက် ပိုမိုကျယ်ပြန့်သော မျိုးစုံမျိုးကွဲပြားမှုနှင့်အတူ၊ လူနှင့်မြွေထိတွေ့နိုင်ခြေမြင့်မားခြင်းနှင့် အများပြည်သူအား ထိထိရောက်ရောက်အသိပညာပေးနိုင်မှု လိုအပ်ချက်များသည် မြန်မာနိုင်ငံအတွင်း မြွေဟောက်ကိုက်ခြင်းကို ထိရောက်စွာ စီမံခန့်ခွဲရန် အဟန့်အတားဖြစ်နေစေပါသည်။ ရပ်ရွာအသိပညာပေးမှုများ၊ အဆိပ်နှင့် အဆိပ်ဖြေဆေးဆိုင်ရာသုတေသနလုပ်ငန်းကို တိုးချဲ့မှုများနှင့် သင့်လျော်သော ဆေးကုသမှုစောင့်ရှောက်မှုကို အချိန်မီရရှိနိုင်စေခြင်းများသည် မြန်မာနိုင်ငံအတွင်းမြွေဟောက်အဆိပ်သင့်ခြင်း၏ ကြီးမားသောဝန်ထုပ်ဝန်ပိုးကို လျှော့ချရန်အတွက် မရှိမဖြစ်လိုအပ်ကြောင်း စာတမ်းတွင်ဖော်ပြထားပါသည်။
