# Supplementary material for: The cobras (genus Naja) of Myanmar: An updated species list with information on identification, distributions, and medical importance
Source: PLoS Negl Trop Dis. 2026 Jun 29;20(6):e0014445. doi: 10.1371/journal.pntd.0014445 (PMC13340773; doi:10.1371/journal.pntd.0014445)
Supplement: Attachment — Submitted filename: PLoS_NTD_reviewer.pdf [file pntd.0014445.s003.pdf]

General comments: The authors present an updated list of the cobras (genus *Naja*) occurring in Myanmar, based on data gathered from iNaturalist and social media. In particular, they currently recognise five cobra species in the country and suggest the presence of a sixth. Furthermore, they compare the distribution of human-cobra encounters throughout the year with average monthly rainfall, and provide an overview of several key issues and guidelines relating to cobra snakebite management and treatment in Myanmar. The study is interesting, the social media-based approach is intriguing, and the findings could prove relevant from both medical and herpetological perspectives. Nonetheless, several issues concerning the interpretation of the analyses conducted, as well as the fact that the World Health Organization has listed for Myanmar some of the species newly reported by the authors since at least 2023, appear substantial and threaten the consistency and novelty of the work. In light of this, in my opinion the manuscript is not acceptable for publication in its current form and requires revision. Further details are provided in the points below.

Line 60 and several other instances across the manuscript: In scientific writing, the term "significance" should only be used in the context of statistical testing. In this work, the authors often use it improperly, to express the concept of "relevance" and/or "importance". The same can be said for the adjective "significant". Please correct these instances across the whole text using more appropriate alternatives.

Line 70: The authors report that 14 ecoregions are currently recognised in Myanmar. This estimate appears not to align with more recent assessments (see https://doi.org/10.1093/biosci/bix014 and https://ecoregions.appspot.com). Please double-check this.

Line 78: As providing a reliable estimate of the number of venomous snake species in Myanmar is challenging, particularly considering the lack of updated information on the country's ophidiofauna, I suggest reporting the number of medically relevant snake species instead. According to the World Health Organization (WHO)'s Snakebite Information and Data Platform, these are 13 (https://snbdatainfo.who.int/?data_id=dataSource_10-187d5a34599-layer-4%3A88).

Line 88: In line with the authors' statement, I believe they should further emphasise that these figures are unlikely to represent antivenom usage patterns across the country as a whole.

Lines 91 and 92: The expression "certain conditions" sounds a bit vague. I suggest the authors be more specific about the conditions to which they are referring.

Line 102: Evidence suggests that classifying *Naja kaouthia* as a strictly non-spitting cobra is likely an oversimplification. Several reports (many of which referenced in the manuscript) describe venom-spitting behaviour in this species, albeit admittedly less proficiently than in some of its congeners. Given that the factors determining the occurrence of spitting behaviour in this species remain unknown, and considering that research specifically focused on cobras in Myanmar is limited (as the authors note in lines 147 and 148), it cannot be ruled out a priori that *N. kaouthia* populations in this country may exhibit spitting behaviour. I

therefore recommend that the authors consider this species as a “potential” spitter rather than a non-spitting cobra.

Lines 113 and 114: This statement appears to be flawed. The WHO's Snakebite Information and Data Platform already recognises *Naja kaouthia*, *Naja mandalayensis*, *Naja siamensis* and *Naja sumatrana* among the 13 snake species of medical relevance present in Myanmar. The development of the platform, including definition of species ranges, was performed in collaboration with some of the world’s leading authorities on snakes and snakebite. The authors should acknowledge that the WHO has recognised *N. siamensis* and *N. sumatrana* as members of the venomous snake fauna of Myanmar since at least 2023. As this likely diminishes the novelty of the work made by the authors, some thorough rephrasing of the whole manuscript should be considered.

Line 132: For the sake of clarity, please specify which "visible morphological/phenotypic characters" the species identifications were based on.

Lines 133 and 134: Details on the additional 32 iNaturalist records are needed. Were they also reviewed by the authors before use? Did they all include verifiable photographic evidence?

Lines 134 and 135: Referring already to “the six species addressed herein” appears premature, as this represents one of the main results of the study. Please rephrase accordingly. Alternatively, the authors could explicitly list the cobra species for which they retrieved data from iNaturalist in Myanmar.

Lines 135–138: Was additional geographic data for *N. kaouthia*, *N. mandalayensis* and *N. sagittifera* from different sources added to achieve a greater number of points for the country? Please specify.

Lines 145 and 146: As already commented, the actual list of the six *Naja* species considered is not provided until Results and Discussion.

Lines 150 and 151: Considering the paucity of data on cobras in Myanmar, and given that “most species of cobra show high geographic variation” (as stated by the authors in the caption to Table 1), on what basis can it be assumed that adult individuals of the six Naja species considered in this study typically resemble those depicted in the photographs provided?

Line 155: Please specify whether the Facebook groups were the same ones as those mentioned before or not.

Lines 159–161: Why did the authors specifically and exclusively focus on rainfall? Several other factors could have been taken into account. Did the authors consider performing some basic modelling including bioclimatic predictors (e.g., temperature, insolation) and/or habitat types?

Lines 161 and 162: The sample sizes do not appear too small to perform basic statistical analysis. Particularly, 91 *N. kaouthia* individuals represent a more than respectable sample size for the average herpetologist!

Lines 166–170: This is the first point in the text at which all six species are explicitly mentioned, although they are implied in earlier sections. As this may be confusing for the reader, I encourage the authors to revise the text accordingly.

Lines 173–175 (Caption to Table 1): The same concerns expressed in the comment to lines 150 and 151 apply.

Table 1: Is *N. sagittifera* merely hypothesised to occur on Coco Island, without any confirmed records? If so, I believe it deserves considerably less space in the manuscript than is currently allocated. Additionally, the authors previously define *N. kaouthia* as non-spitting, yet in the table its potential spitting behaviour is acknowledged, albeit considered rare. I recommend amending the main text for consistency (see comment on line 102). Finally, when reporting the maximum size of each species, please provide references (as done in the species accounts) and clarify whether the measurement refers to maximum snout–vent length (SVL) or maximum total length (SVL + tail length).

Figure 1: I recommend reorganising the country names, in order to avoid them not fitting in one line and/or getting confused with country borders. Adjusting or reducing the font size should be sufficient.

Lines 181–184 (Caption to Figure 1): I suggest changing *N.* sp. to *Naja* sp. (also in the figure legend). Furthermore, the line drawing of *N. kaouthia* seems superfluous, and does not convey any relevant information.

Line 188: I suggest changing "Species of Known Occurrence" to something like "Historically Documented Species".

Line 227: Although traditionally considered a non-spitting cobra, current evidence indicates that *N. kaouthia* does spit venom in some parts of its distribution. Exactly as stated in lines 229 and 230, the occurrence of venom-spitting behaviour in this species in Myanmar cannot be ruled out a priori.

Line 233: It is worth including Rahman et al., 2025 (https://doi.org/10.1016/j.toxicon.2024.108221) among the references regarding venom ophthalmia caused by *N. kaouthia* venom.

Lines 233–235: Please reference the official WHO guidelines for the management of snakebites (https://www.who.int/publications/i/item/9789290225300; page 164) and Chu et al., 2010 (https://doi.org/10.1016/j.toxicon.2010.02.023).

Lines 263–266: Correctly interpreting the methodological approach of Neto et al. on the proteomics of *N. mandalayensis* venom is admittedly challenging. It is my understanding that the information presented in the pie charts does not represent an actual quantification of the

relative abundances of the toxins in *N. mandalayensis* venom, but rather reflects their diversity. I suggest that the authors consider rephrasing this section accordingly.

Lines 272–274: Unless this traditional remedy has been demonstrated to have any medical efficacy, I recommend that the authors clearly state that its use should be discouraged in favour of the standard snakebite treatment and management procedures endorsed by the World Health Organization.

Lines 321–323: It may be worth mentioning that *N. fuxi* was previously considered *N. kaouthia*, which is recognised as Category 1 throughout its entire range in Myanmar.

Lines 328–331: A similar situation applies to *N. kaouthia*, within which *N. fuxi* was originally classified.

Lines 420–422: As already mentioned, the WHO Snakebite Information and Data Platform actually already lists *N. sumatrana* (as well as *N. siamensis*) among the medically relevant snake species of Myanmar.

Line 486: Given the scarcity of behavioural data for most of the *Naja* species discussed here, I recommend adopting this more cautious phrasing also when referring to the (putative) "non-spitters" of Myanmar.

Lines 495 and 496: I suggest explicitly naming the species to which the authors are referring, in order to avoid potential confusion.

Lines 496–500: In my view, this section requires critical reconsideration, as the interpretation presented has several substantial limitations. First, the authors compare opportunistic observations with records derived from active searches. As these two data sources are not methodologically comparable, this appears to represents a classic "apples and oranges" problem. Moreover, encounter frequency alone cannot be assumed to reflect snake activity, and it is not possible to disentangle true biological patterns from variation in detection probability without correcting for sampling effort. The authors also treat the Thailand dataset as a biological baseline, although it appears to be another human-mediated dataset. Interpreting this pattern as biologically representative is not justified without independent validation.

Lines 501–509: Have the authors considered the possibility that the high proportion of encounters reported near human habitation reflects observer distribution rather than cobra distribution? Most observations may have occurred near homes because most observers were located there, not because cobras are disproportionately associated with human habitation.

Lines 510–516: Assuming that encounter frequency predicts snakebite incidence likely constitutes an inference. Furthermore, agricultural and forestry activities in Myanmar are seasonal. As most bites occur during agricultural or forest-related work, exposure risk is

expected to vary seasonally. In this context, the seasonality of snakebite may reflect work cycles rather than cobra visibility near homes.

Line 527: The World Health Organization lists Myanmar Pharmaceutical Factory as a domestic producer. Is this the same producer mentioned here?

Line 535: Here are a couple more recent references worth considering: https://doi.org/10.1371/journal.pntd.0000569; https://doi.org/10.1038/nrdp.2017.63

Line 553: I encourage the authors to clarify what they mean by “less harmful” methods. The traditional remedies listed here still delay the application of effective treatments, potentially increasing the severity of envenomation symptoms.

Lines 608–612: These considerations should be highlighted also when mentioning the relevance of traditional remedies.

Line 650: Is Myanmar Pharmaceutical Factory an alternative name for the Burmese Pharmaceutical Industries mentioned before?

Lines 700 and 701: Fasciotomy is not only not recommended, but is also often unnecessary when appropriate antivenom is administered successfully.

Lines 706–708: I suggest mentioning *N. kaouthia* from Myanmar as a potential spitter.

Abstract and Conclusion: In light of the points raised above and the amendments required, these two sections should be revised.
