# Supplementary material for: The cobras (genus Naja) of Myanmar: An updated species list with information on identification, distributions, and medical importance
Source: PLoS Negl Trop Dis. 2026 Jun 29;20(6):e0014445. doi: 10.1371/journal.pntd.0014445 (PMC13340773; doi:10.1371/journal.pntd.0014445)
Supplement: Attachment — Submitted filename: Responses to Reviewers.docx [file pntd.0014445.s004.docx]

**Responses to Reviewers:**

Dear Editor,

We are grateful to Reviewers #1, #2 and #4 for the time they have taken to help us improve our manuscript. We outline below the changes we have made in response to their concerns.

The comments of Reviewer #3 are voluminous but highly unspecific and often peripherally relevant at best. This review shows all the hallmarks of having written by GenAI software rather than a human reviewer. On that basis, and since we have three constructive reviews from clearly human reviewers, we have taken the liberty of not engaging with the comments of Reviewer #3

We look forward to hearing from youin due course.

On behalf of all authors,

Yours sincerely,

Wolfgang Wüster
Professor of Zoology (Herpetology)

Bangor University

**Methods**


-Are the objectives of the study clearly articulated with a clear testable hypothesis stated?

-Is the study design appropriate to address the stated objectives?

-Is the population clearly described and appropriate for the hypothesis being tested?

-Is the sample size sufficient to ensure adequate power to address the hypothesis being tested?

-Were correct statistical analysis used to support conclusions?

-Are there concerns about ethical or regulatory requirements being met?

Reviewer #1: The methods are well described and adequate for the type of study. Using citizen science data is a useful approach for filling knowledge gaps in crisis disciplines, such as snakebite mitigation, where additional data is urgently needed but difficult to obtain through more conventional data collection approaches. Any limitations associated with this approach are clearly discussed in the manuscript.

We thank the reviewer for these comments.

Reviewer #2: Details provided in the attached PDF file.

Comments addressed individually at the end of this letter.

Reviewer #3: This manuscript aims to clarify which Naja cobra species occur in Myanmar, update their distributions using a synthesis of museum/literature evidence and vetted photographic (citizen-science/social media) records, and discuss implications for snakebite management. The objectives are generally clear for a synthesis paper, but the manuscript would benefit from sharper, explicitly stated, testable claims that align with the evidence assembled—e.g., “Species X occurs in Myanmar,” “Species Y is restricted to region Z,” or “reported encounters peak in months A–B.” As written, some statements read as definitive confirmation even where the underlying evidence appears sparse. For acceptance, the manuscript should consistently differentiate what is directly demonstrated by vouchered specimens or clearly diagnostic, traceable photographic records from what is probable or possible based on limited or lower-confidence reports. This is essential to ensure the conclusions are accurately reported and scaled to the strength of evidence.

The overall study design is appropriate for the stated aims. A mixed-source occurrence synthesis is a reasonable approach in settings where systematic survey coverage is limited. However, to meet acceptance criteria, the design must be reported with sufficient transparency that another team could replicate the record-assembly and vetting workflow and reach comparable inferences. In particular, the manuscript should explicitly describe the universe of sources searched (platforms, museum collections, and literature scope), the time window covered, inclusion/exclusion rules, how duplicates were handled, how locality information was georeferenced (and how uncertain localities were treated), and how identification decisions were made and adjudicated when ambiguous. Because citizen-science/social-media observations are inherently biased toward accessible locations, settlements, and user participation, the Methods should explicitly acknowledge this bias and the Results/Discussion should interpret “seasonality” as reported encounter seasonality rather than true cobra activity seasonality unless independent evidence supports biological inference.

In this work, the relevant “population” is the set of cobra occurrence records in Myanmar, not human participants. That population needs clearer operational definition—what constitutes a Myanmar record when locality precision varies, and what precision thresholds are required for inclusion in distribution mapping. The adequacy of sample size is uneven across taxa: where taxa are supported by multiple independent records and/or vouchers, descriptive distribution updates are plausible. Where taxa are supported by very few records (possibly a single record), this is still valuable, but it cannot sustain strong claims about broad distribution, stable seasonal patterns, or downstream clinical implications without careful qualification. Acceptance therefore depends on aligning the strength of statements with record volume and confidence: sparse-record taxa should be framed as probable/possible unless supported by high-tier evidence, and any temporal inference should be restricted to records with reliable date metadata.

The statistical components appear largely descriptive, which is appropriate for a synthesis paper, but the key issue is interpretive validity rather than choice of tests. Any seasonal patterns derived from opportunistic reporting should be presented cautiously and accompanied by either a simple robustness check or clearly stated limitations. For acceptance, the authors should either (i) provide a minimal sensitivity analysis repeating the seasonal plot using only high-confidence records with precise dates and locations and stating whether the pattern persists, or (ii) substantially rewrite the seasonality section so it remains descriptive and does not imply ecological causation. Similarly, medical/antivenom statements require careful calibration: any claim about antivenom efficacy or cross-neutralization must be tied explicitly to the type of evidence available (clinical vs preclinical vs inference). Where direct evidence is absent, the manuscript should use conditional language and frame recommendations as priorities for evaluation rather than implied proven adequacy or inadequacy.

Ethical and regulatory considerations are limited because there is no direct human-subject involvement. However, publication ethics transparency is still essential because the manuscript uses social-media images and in some cases observer communications. For acceptance, the manuscript should include a concise ethics/permissions statement covering: permissions and crediting for photographs used in figures, anonymization (no personally identifying information disclosed), and how sensitive locality information is handled (e.g., avoiding unnecessary precision where it could create risk). These are typically straightforward additions but important for compliance and reader trust.

In practical terms, the essential work required for acceptance is achievable within a short revision cycle and does not require new field collections. The authors should implement a clear evidence-tier framework (voucher/diagnostic photo/probable/possible) and apply it consistently across tables, species accounts, abstract, and conclusions; expand Methods to make the citizen-science workflow reproducible (sources searched, search strategy, screening criteria, duplicate rules, georeferencing, identification adjudication); add uncertainty flags to the supplementary dataset (ID confidence, locality precision, record type, diagnostic visibility); address seasonality interpretation through either a simple robustness check or a rewrite that emphasizes reporting bias; tighten all antivenom/medical claims so they strictly reflect available evidence; and include an explicit ethics/permissions statement for social-media content. With these targeted revisions, the manuscript would more reliably communicate what the data show, transparently convey limitations, and provide a reproducible, conservative update of Myanmar’s Naja cobra composition and distribution with appropriately framed public-health implications.

See above - this is almost certainly an AI-generated review.

Reviewer #4: No comments or concerns.

We thank the reviewer for these comments.

**Results**


-Does the analysis presented match the analysis plan?

-Are the results clearly and completely presented?

-Are the figures (Tables, Images) of sufficient quality for clarity?

Reviewer #1: The results appropriately summarize the collected data and are clearly presented.

We thank the reviewer for these comments.

Reviewer #2: Details provided in the attached PDF file.

Comments addressed individually at the end of this letter.

Reviewer #3: The Results section is broadly aligned with the manuscript’s intended workflow—assembling and vetting occurrence records from multiple sources and presenting the outcomes as tables, maps, and descriptive summaries. However, acceptance will depend on making the Results more transparently traceable to the analysis plan, more complete in reporting the evidence base behind each conclusion, and clearer and more publication-ready in its figures and tables.

The findings reflect a compilation-and-synthesis approach consistent with the stated aims. Where the match is currently weakest is in demonstrating the outputs of key curation steps that are implied in the Methods (screening, deduplication, georeferencing, identification adjudication, and confidence assessment). To show clear alignment with the analysis plan, the Results should report at least minimal summary counts—how many records were identified, how many removed as duplicates, how many excluded for insufficient diagnostic quality or uncertain locality/date, and how many retained per species and per record type. Without these counts, it is difficult for readers to evaluate whether the presented distributions and species status statements arise from a structured analysis plan or from an unquantified selection process.

The findings are also generally understandable, but they are not yet complete in the sense that a reader can verify the strength of evidence behind each major claim. For acceptance, the Results should consistently quantify the evidence supporting each species’ status and distribution: number of retained records per species, broken down by record type (voucher specimen, diagnostic photograph, literature-only), and—if you adopt the recommended approach—by an explicit confidence/evidence tier and locality precision category. This is especially important for taxa supported by very few records; those results remain valuable, but the narrative must clearly reflect the limited sample size and should avoid definitive phrasing unless supported by high-tier evidence. In addition, temporal/seasonality results should be framed explicitly as reported encounter patterns and should present sample sizes so that readers can judge robustness; if feasible, a brief sensitivity statement using high-confidence records only would further strengthen completeness and prevent overinterpretation.

The figures and tables are central to this manuscript, but several need improvement to meet publication standards for clarity and self-contained interpretation. Key tables should define evidence categories and abbreviations in footnotes and include record counts per species by type and confidence. Maps should use consistent symbology across panels, include clear legends, and remain readable at journal print size; they should also clarify what locality precision is shown and caution against interpreting point density as abundance. Photographic figures (if included) should show diagnostic traits clearly, include proper credits/permissions, avoid revealing sensitive information, and use captions that state what the image demonstrates diagnostically. Seasonal and summary plots should clearly label axes/units, include sample sizes, and avoid visual designs that imply causation (e.g., rainfall overlays) unless the text and caption explicitly treat them as descriptive comparisons.

For the results to be acceptable, the authors should: (1) add a consolidated Results table listing, for each species, the total number of retained records and the breakdown by voucher/diagnostic photo/literature-only (and ideally by confidence tier and locality precision); (2) include a short screening/curation summary (records found → duplicates removed → excluded → retained) to demonstrate the Results match the analysis plan; (3) rewrite Results statements so that “confirmed/probable/possible” wording strictly matches the evidence tier and record volume; (4) treat seasonality explicitly as reported encounters, report sample sizes, and either add a simple high-confidence sensitivity note or keep interpretation strictly descriptive; and (5) strengthen captions so every figure/table is standalone, legible at publication size, and clear about what can and cannot be concluded. These changes do not require new fieldwork but are essential to ensure the Results are accurately reported and interpretable.

See above - this is almost certainly an AI-generated review.

Reviewer #4: Overall the results are clearly presented. Some figures lack appropriate photographer credit within the captions, and this should be updated.

We thank the reviewer for these comments. We have adjusted photographer credits for all images.

**Conclusions**


-Are the conclusions supported by the data presented?

-Are the limitations of analysis clearly described?

-Do the authors discuss how these data can be helpful to advance our understanding of the topic under study?

-Is public health relevance addressed?

Reviewer #1: The conclusions correctly reflect the results, are well presented, and address any limitations of the study. My only concern would be that the elaborate discussion of snakebite management is not directly related to the collected data but is more of a review. However, I agree that the inclusion of these sections is a very useful addition because it makes all information on the topic of cobras and their bites in Myanmar easily accessible for readers in one manuscript.

We thank the reviewer for these comments and agree that while the discussion of snakebite is not based on the data presented within it is an important resource warranting inclusion.

Reviewer #2: Details provided in the attached PDF file.

Comments addressed individually at the end of this letter.

Reviewer #3: The conclusions are broadly consistent with the manuscript’s purpose—providing an updated synthesis of Naja species occurrence and distribution in Myanmar with implications for snakebite management—but several statements need to be recalibrated so they strictly reflect what the compiled evidence supports. For acceptance, definitive wording such as “confirmed presence,” strong distribution claims, or statements implying robust seasonality should be reserved for taxa and patterns supported by high-confidence evidence (vouchered specimens or clearly diagnostic, traceable records). Where evidence is sparse or opportunistic, conclusions should be phrased as “supported by limited records,” “probable,” or “requiring confirmation,” and temporal patterns should be described as reported encounter patterns rather than biological seasonality unless independently validated.

The limitations are not yet stated clearly enough in the concluding section. A short, explicit limitations paragraph should be added immediately before (or within) the Conclusions to constrain interpretation. This should include: strong reporting/sampling bias in citizen-science sources, uneven geographic coverage, variable locality/date precision, possible misidentification from non-diagnostic images, and small sample sizes for some taxa. Where the manuscript draws medical implications, it should also plainly note limits to inference about antivenom performance (e.g., geographically structured venom variation and limited direct neutralization/clinical evidence for some taxa).

The manuscript can advance understanding in practical and defensible ways, and the Conclusions should state these more concretely: it provides a clearer, evidence-based national picture of cobra species composition and distribution; it identifies priority geographic and taxonomic gaps for targeted field surveys and voucher-based confirmation; and it offers a rational basis for prioritizing venom sampling and antivenom cross-neutralization testing.

Public health relevance is appropriately raised, but because this is high-stakes, the closing statements should be framed cautiously and operationally. Rather than implying proven antivenom efficacy or failure where direct evidence is not presented, the Conclusions should emphasize what the data allow: improved clarity on likely offending species distributions, better targeting of identification and training resources, and explicit priorities for evaluating antivenom coverage and strengthening surveillance. With these targeted revisions, the Conclusions will be evidence-aligned, transparent about uncertainty, and more useful for both biodiversity and snakebite stakeholders.

See above - this is almost certainly an AI-generated review.

Reviewer #4: No comments or corrections. The data and results presented are of significant public health relevance.

We thank the reviewer for these comments.

**Editorial and Data Presentation Modifications?**


Use this section for editorial suggestions as well as relatively minor modifications of existing data that would enhance clarity. If the only modifications needed are minor and/or editorial, you may wish to recommend “Minor Revision” or “Accept”.

Reviewer #1: L 428: reference (Leong et al., 2015) is not formatted correctly,

We thank the reviewer for catching this formatting error and have corrected this mistake.

Reviewer #2: Details provided in the attached PDF file.

Comments addressed individually at the end of this letter.

Reviewer #3: The manuscript would benefit from several editorial and data-presentation refinements that do not require new data collection but would substantially improve clarity, traceability, and reader confidence.

First, improve consistency and precision of terminology throughout. Use one standard set of terms for records (e.g., “voucher specimen,” “diagnostic photograph,” “literature record,” “unverified report”) and apply them consistently in the text, tables, and captions. Avoid mixing “confirmed/verified/recorded” interchangeably unless you define each term and use it systematically. Where you discuss temporal patterns, use “reported encounters/observations” rather than “activity” unless you explicitly justify biological inference.

Second, strengthen figure and table self-containment. Key tables should include clear footnotes defining abbreviations, evidence categories, and any confidence classes. Where you summarise species presence and distribution, include basic counts (number of retained records by type and confidence tier) directly in the main table rather than leaving these details implicit. Even if you already have these in supplementary files, a compact main-table version makes the paper much easier to interpret.

Third, improve map readability and comparability. Ensure consistent symbology across panels, legible administrative labels at journal print size, and standardized legends. If multiple maps are presented, standardize scale bars and north arrows. Consider using distinct symbols for record types (e.g., voucher vs photo) and, if you include uncertainty, a simple visual cue for locality precision (exact vs approximate). Add a short note in the caption cautioning that point density reflects reporting effort as well as occurrence.

Fourth, refine photographic figure presentation (if included). Ensure all photos used in figures have explicit credits and permission statements as required, highlight diagnostic features (e.g., with unobtrusive arrows or inset zooms if allowed by the journal), and avoid any content that reveals sensitive personal information or overly precise sensitive localities.

Fifth, tidy Results/Discussion structure to reduce repetition and improve flow. Use consistent subheadings that separate (i) species occurrence/distribution updates, (ii) encounter timing/seasonality, and (iii) medical/public health implications. Keep Results descriptive and move mechanistic explanations into Discussion to avoid over-interpretation.

Sixth, ensure the supplementary dataset is clean and maximally useful. Include standardized fields such as record source, record type, date, locality description, coordinates, coordinate uncertainty/precision class, identification confidence, and whether a voucher/photo exists. Check for duplicate rows, inconsistent place naming, and missing units. A brief “data dictionary” paragraph (or table) in the supplement would further improve usability.

See above - this is almost certainly an AI-generated review.

Reviewer #4: Discussed in summary.

Addressed in summary.

**Summary and General Comments**


Use this section to provide overall comments, discuss strengths/weaknesses of the study, novelty, significance, general execution and scholarship. You may also include additional comments for the author, including concerns about dual publication, research ethics, or publication ethics. If requesting major revision, please articulate the new experiments that are needed.

Reviewer #1: The manuscript thoroughly reviews available data on cobra bite management in Myanmar and analyses important new data on cobra distributions and available antivenoms. Venomous snake distributions are often insufficiently understood and clarifying them is crucial for adequate snakebite management, since snakes are often not identifiable by victims and treatment needs to be decided on based on which snakes are present in the region.

Hi Anna — thanks very much for your kind appraisal of our work! We are very appreciative of your thoughtful and constructive review. Cheers, Neil.

Reviewer #2: Details provided in the attached PDF file.

Comments addressed individually at the end of this letter.

Reviewer #3: This manuscript provides a timely synthesis of Myanmar’s Naja cobras by combining multiple evidence streams (museum/literature sources and vetted photographic/citizen-science records) to update species composition, distribution patterns, and the likely implications for snakebite management. The main strength is its practical intent: clarifying which cobra taxa are present and where they occur is valuable for clinicians, public health planning, and biodiversity monitoring. The work also demonstrates strong scholarship in assembling dispersed information into a single narrative and presenting it in a way that can be operationally useful.

The main weakness is not the idea, but the calibration of claims to evidence strength. Some summary statements read as definitive confirmations even where the underlying record base appears sparse or derived from opportunistic reporting with variable metadata quality. This matters because the paper’s conclusions are likely to be used beyond herpetology (e.g., in clinical contexts), where overstated certainty can mislead. A second weakness is reproducibility: the record-vetting and georeferencing workflow is described in general terms, but key details are missing (search strategy, inclusion/exclusion rules, duplicate handling, confidence assignment, and identification adjudication). Without these, readers cannot fully evaluate bias and cannot replicate the dataset assembly.

Novelty is moderate but meaningful for the target context: the manuscript’s value lies less in a new analytical method and more in an updated, evidence-informed national synthesis that integrates underused citizen-science observations with curated records. The public health significance is potentially high, but only if the medical statements are framed conservatively and explicitly tied to the type of evidence available (clinical vs preclinical vs inference), and if limitations (reporting bias, uneven coverage, locality/date uncertainty, and sparse records for some taxa) are clearly stated and used to bound conclusions.

No clear dual-publication concerns are apparent from the manuscript as reviewed, but publication ethics requirements should be handled explicitly given the use of social-media photographs: permissions/crediting, observer privacy/anonymization, and handling of sensitive locality information should be clearly stated. There are no apparent ethical concerns involving human participants, but the paper should confirm that any private communications were used with consent and that personal identifiers are not disclosed.

The essential “information requested does not need to be new experiments in the laboratory or new field collections. Rather, it should be limited, feasible, and focused on ensuring the results are accurately reported and reproducible: (1) implement an explicit evidence-tier framework (voucher/diagnostic photo/probable/possible) and apply it consistently across the abstract, tables, species accounts, and conclusions; (2) provide a fully reproducible description of the citizen-science record acquisition and vetting workflow (platforms searched, search terms, date range, inclusion/exclusion criteria, duplicate rules, georeferencing method, and ID adjudication); (3) include confidence and locality-precision flags in the supplementary occurrence dataset; and (4) either add a simple robustness check for the seasonality pattern using high-confidence records or rewrite that section to remain strictly descriptive of reported encounters. These revisions should be achievable within a short revision cycle and would substantially strengthen credibility, interpretability, and public health utility.

See above - this is almost certainly an AI-generated review.

Reviewer #4: In this manuscript Balchan and colleagues review and update the current knowledge on the distribution, identification, medical relevance and overall envonoming treatment of medically significant cobras (Naja spp) in Myanmar. I found the study to be quite thorough and informative, including relevant natural history and distributional data for all species. I have one minor suggestion that I think is relatively important to address, that will aid in the overall usefulness of this paper as a tool for cobra identification within Myanmar. Although the authors are experts on snake identification and they do explicitly discuss morphological characters used to distinguish among the species in question, often these characters overlap, and the important characters (such as hood markings) are not always shown in their figures illustrative of "typical" examples of the species within the region (Figs 2-6). My suggestion is that the authors update these figures to include 1) insets of hood markings (either illustration or photos) typically associated with each species, and 2) a description in the caption of exactly which characters were used to determine the IDs of the specific individuals in the photos. This way readers will have an easier grasp on how IDs were determined.

Otherwise I found the manuscript to be quite a fine and important addition to the literature of medically relevant snakes in this understudied region.

We thank the reviewer for these kind comments and positive appraisal of our work. We have adjusted the indicated figures so that 1) each includes an inset illustration of typical hood markings and 2) captions clarify which features were used to determine species identities.

Comments from Reviewer #2:

General comments: The authors present an updated list of the cobras (genus Naja) occurring in Myanmar, based on data gathered from iNaturalist and social media. In particular, they currently recognise five cobra species in the country and suggest the presence of a sixth. Furthermore, they compare the distribution of human-cobra encounters throughout the year with average monthly rainfall, and provide an overview of several key issues and guidelines relating to cobra snakebite management and treatment in Myanmar. The study is interesting, the social media-based approach is intriguing, and the findings could prove relevant from both medical and herpetological perspectives. Nonetheless, several issues concerning the interpretation of the analyses conducted, as well as the fact that the World Health Organization has listed for Myanmar some of the species newly reported by the authors since at least 2023, appear substantial and threaten the consistency and novelty of the work. In light of this, in my opinion the manuscript is not acceptable for publication in its current form and requires revision. Further details are provided in the points below.

We thank the reviewer for these comments and for raising points about WHO recognition of some cobra species since 2023 — we were unaware of this. We have adjusted the manuscript to reflect this information, and these edits are detailed below in response to individual comments.

Line 60 and several other instances across the manuscript: In scientific writing, the term "significance" should only be used in the context of statistical testing. In this work, the authors often use it improperly, to express the concept of "relevance" and/or "importance". The same can be said for the adjective "significant". Please correct these instances across the whole text using more appropriate alternatives.

We have corrected our usage of this word in 11 instances throughout the text, and the terms “medically significant” or “medical significance” are no longer used within. We thank the reviewer for raising this point.

Line 70: The authors report that 14 ecoregions are currently recognised in Myanmar. This estimate appears not to align with more recent assessments (see https://doi.org/10.1093/biosci/bix014 and https://ecoregions.appspot.com). Please double-check this.

We have double checked out figure against the indicated source. The more recent assessment lists the same 14 ecoregions for mainland Myanmar: Irrawaddy moist deciduous forests, Irrawaddy dry forests, Irrawaddy freshwater swamp forests, Myanmar coastal rain forests, Myanmar coastal mangroves, Mizoram–Manipur–Kachin rain forests, Kayah–Karen montane rain forests, Tenasserim–South Thailand semi-evergreen rain forests, Tenasserim–South Thailand moist deciduous forests, Northern Indochina subtropical forests, Northern Triangle temperate forests, Nujiang Langcang Gorge alpine conifer and mixed forests, Eastern Himalayan alpine shrub and meadows, and Chin Hills–Arakan Yoma montane forests.

It does differ in including the Andaman Islands rain forests ecoregion for the Coco Islands as a 15th ecoregion. We have changed the number of ecoregions to 15 (line 84) and added a citation to this more recent assessment in addition to our original source.

Line 78: As providing a reliable estimate of the number of venomous snake species in Myanmar is challenging, particularly considering the lack of updated information on the country's ophidiofauna, I suggest reporting the number of medically relevant snake species instead. According to the World Health Organization (WHO)'s Snakebite Information and Data Platform, these are 13 (<https://snbdatainfo.who.int/?data_id=dataSource_10-187d5a34599-> layer-4%3A88).

We have modified this sentence (starting line 95) as follows: “At least 39 venomous snake species are currently known from Myanmar [1] with 19 species designated as medically relevant by the World Health Organization [6], although this number is likely a considerable underestimate pending further taxonomic work [7,8] and updated distributional data [9].”

Line 88: In line with the authors’ statement, I believe they should further emphasise that these figures are unlikely to represent antivenom usage patterns across the country as a whole.

We have modified the indicated sentence as follows: “Data from Mandalay General Hospital indicate substantially greater usage of the BPI Viper Antivenom (8,848 vials) compared to the BPI Cobra Antivenom (30 vials) over the twelve-month study period [10], although these figures are unlikely to represent antivenom usage patterns across the country as a whole.”

Lines 91 and 92: The expression “certain conditions” sounds a bit vague. I suggest the authors be more specific about the conditions to which they are referring.

We have modified this sentence to read: “although other studies find partial or effective neutralization despite venom–antivenom mismatch [14].”

Line 102: Evidence suggests that classifying Naja kaouthia as a strictly non-spitting cobra is likely an oversimplification. Several reports (many of which referenced in the manuscript) describe venom-spitting behaviour in this species, albeit admittedly less proficiently than in some of its congeners. Given that the factors determining the occurrence of spitting behaviour in this species remain unknown, and considering that research specifically focused on cobras in Myanmar is limited (as the authors note in lines 147 and 148), it cannot be ruled out a priori that N. kaouthia populations in this country may exhibit spitting behaviour. I therefore recommend that the authors consider this species as a “potential” spitter rather than a non-spitting cobra.

We dive into this topic more deeply starting at line 288. At present, no published records indicate venom spitting from Burmese *N. kaouthia*, and the authors have not run across any examples of this (whether anecdotal, during our own fieldwork, word of mouth, or otherwise). We go into detail on the geographic prevalence of venom spitting in *N. kaouthia* in Balchan et al. 2025 (10.11646/zootaxa.5717.4.2). While we don’t rule out venom spitting as a possibility for Burmese *N. kaouthia* and are aware that it is prevalent in adjacent Bangladesh and India, the overwhelming lack of evidence for venom spitting across the Burmese range of this species justifies our classification of it as a “non-spitting” cobra for the sake of simplicity for the reader. We do temper this with a statement in the *N. kaouthia* species account (line 243): “Although venom spitting has not been reported from Myanmar, its occurrence, particularly in western populations, cannot be ruled out.”

Lines 113 and 114: This statement appears to be flawed. The WHO's Snakebite Information and Data Platform already recognises Naja kaouthia, Naja mandalayensis, Naja siamensis and Naja sumatrana among the 13 snake species of medical relevance present in Myanmar. The development of the platform, including definition of species ranges, was performed in collaboration with some of the world’s leading authorities on snakes and snakebite. The authors should acknowledge that the WHO has recognised N. siamensis and N. sumatrana as members of the venomous snake fauna of Myanmar since at least 2023. As this likely diminishes the novelty of the work made by the authors, some thorough rephrasing of the whole manuscript should be considered.

We were unaware of this information and have added this to the manuscript (Lines 133-137).

We retrieved the occurrence data for *N. siamensis* and *N. sumatrana* used in the WHO maps (via David Williams) to confirm these observations. A single observation is present for *N. siamensis* in Tachilek, Myanmar (iNaturalist 145428671) – this same record is included in our paper, in addition to several others that expand the distribution in Myanmar. A single observation for *N. sumatrana* in the WHO dataset places the range of this species in Myanmar, but this record appears to be in error. The included data in the spreadsheet list coordinates that plot in Myanmar, yet the locality data are listed as ‘Thailand”. This record was sourced from a modelling study by Choudhury et al. who sourced it from Grismer et al.’s Red List Assessment. It appears that Choudhury et al. made an error in their coordinate data, as Grismer et al. does not depict the distribution of *N. sumatrana* north of Phangnga and Surat Thani Provinces in Thailand. Consequently, this record is in error, and it seems reasonable that this resulted from Choudhury incorrectly reporting coordinates. No prior records exist to our knowledge of *N. sumatrana* in Myanmar.

The WHO occurrence data are not published anywhere to our knowledge, and while a few records of *N. siamensis* are present on iNaturalist, there is no available published resource affirming the occurrence of either species in the country and detailing voucher photographs and distributional information. We do maintain the perspective that only two cobra species “are widely recognized as occurring in Myanmar” given the status of the current literature and distributional resources and believe that the reporting of confirmed records for both *N. siamensis* and *N. sumatrana* remain a novel aspect of our paper.

Line 132: For the sake of clarity, please specify which "visible morphological/phenotypic characters" the species identifications were based on.

We have modified this sentence (line 159) as follows: “based on visible morphological/phenotypic characters (i.e., scalation, hood markings, body pattern):”

Lines 133 and 134: Details on the additional 32 iNaturalist records are needed. Were they also reviewed by the authors before use? Did they all include verifiable photographic evidence?

We have added a sentence (line 166) indicating this: “All iNaturalist records were reviewed by the authors (NRB and WPO) and contained photographs from which species identifications could be verified.”.

Lines 134 and 135: Referring already to “the six species addressed herein” appears premature, as this represents one of the main results of the study. Please rephrase accordingly. Alternatively, the authors could explicitly list the cobra species for which they retrieved data from iNaturalist in Myanmar.

We have modified this sentence to explicitly state the species for which records were retrieved, as follows:
“An additional 32 records were obtained from iNaturalist on 23 August 2025 encompassing *N. fuxi* (n = 9), *N. kaouthia* (n = 11), *N. mandalayensis* (n = 4), *N. sagittifera* (n = 4), *N. siamensis* (n = 3), and *N. sumatrana* (n = 1) occurring within or near the border of Myanmar.”

Lines 135–138: Was additional geographic data for N. kaouthia, N. mandalayensis and N. sagittifera from different sources added to achieve a greater number of points for the country? Please specify.

We have modified this sentence as follows to specify this:
“Geographic data were also included for specimens of *N. kaouthia* (n = 21) and *N. mandalayensis* (n = 30) held in the collections of the California Academy of Sciences, and six additional points for *N. sagittifera* were included from a previously published study [24] to better approximate ranges for these three species via a greater number of occurrence points.”

Lines 145 and 146: As already commented, the actual list of the six Naja species considered is not provided until Results and Discussion.

We have modified this sentence as follows:
“We provide accounts for six *Naja* species with confirmed or potential occurrence in Myanmar: *Naja fuxi*, *N. kaouthia*, *N. mandalayensis*, *N. sagittifera*, *N. siamensis*, and *N. sumatrana*.”

Lines 150 and 151: Considering the paucity of data on cobras in Myanmar, and given that “most species of cobra show high geographic variation” (as stated by the authors in the caption to Table 1), on what basis can it be assumed that adult individuals of the six Naja species considered in this study typically resemble those depicted in the photographs provided?

Much of this variation is geographically structured for species with high variation – i.e. *N. siamensis* may is typically unicolor olive across Myanmar, northern Thailand, and much of Cambodia, Vietnam, and Laos, but is typically white with black markings in central Thailand, and is unicolor black in its SW range extremity in Thailand. While Myanmar is a large country and there is still much to be uncovered regarding cobra diversity here, the selected images are expected to be representative of the respective species across most of its Myanmar range (based on our inspection of observation photos and specimens), though some level of phenotypic variation should be expected.

Line 155: Please specify whether the Facebook groups were the same ones as those mentioned before or not.

We have added this information, modifying this line as follows:
“To explore seasonal patterns in human–cobra interactions, we analyzed a subset of occurrence records (“Native Species Conservation and Identification in Myanmar” and “Myanmar Snakes” Facebook groups and iNaturalist)”

Lines 159–161: Why did the authors specifically and exclusively focus on rainfall? Several other factors could have been taken into account. Did the authors consider performing some basic modelling including bioclimatic predictors (e.g., temperature, insolation) and/or habitat types?

We understand that a lot more could have been doing with these data but given the quality of metadata associated with Facebook records (often location specified only to a city or region), and biases associated with these observations, we refrained from doing any modelling. A much more detailed analyses of human-snake encounters across a large area of Asia would be a valuable contribution to the literature, but our data don’t nicely lend themselves to this type of project and this was outside of the scope for what we hoped to achieve with this study. The presentation of these data was intended to be largely illustrative. We only focused on rainfall (again from an illustrative perspective) as this is known to be a major driver of snake activity cycles in the tropics.

Lines 161 and 162: The sample sizes do not appear too small to perform basic statistical analysis. Particularly, 91 N. kaouthia individuals represent a more than respectable sample size for the average herpetologist!

We have added several analyses as suggested, these are detailed in Methods (Lines 198-208) and discussed in Human-Cobra Encounters (starting line 593).

Lines 166–170: This is the first point in the text at which all six species are explicitly mentioned, although they are implied in earlier sections. As this may be confusing for the reader, I encourage the authors to revise the text accordingly.

We have modified the prior sections as indicated above so that this now makes sense as written.

Lines 173–175 (Caption to Table 1): The same concerns expressed in the comment to lines 150 and 151 apply.

Please see response above regarding points on variation. Ventral scale counts offer a relatively definitive means of differentiation for many species.

Table 1: Is *N. sagittifera* merely hypothesised to occur on Coco Island, without any confirmed records? If so, I believe it deserves considerably less space in the manuscript than is currently allocated. Additionally, the authors previously define N. kaouthia as non-spitting, yet in the table its potential spitting behaviour is acknowledged, albeit considered rare. I recommend amending the main text for consistency (see comment on line 102). Finally, when reporting the maximum size of each species, please provide references (as done in the species accounts) and clarify whether the measurement refers to maximum snout–vent length (SVL) or maximum total length (SVL + tail length).

We have added references for body length estimates in this table and adjusted wording to indicate “Maximum Total Length”. Please see other comments for additional dialogue on spitting in *N. kaouthia*, particularly our discussion regarding the lack of records of venom spitting in Myanmar in reference to your comment below regarding Lines 328–331. The presence of *N. sagittifera* is only hypothesized based on proximity and occurrence of other Andaman endemics in the Coco Islands. We feel that is important to include information for this species in a similar fashion as we have done for the other species for several reasons. Firstly, the literature on this species is sparse and spread out, so our summary provides a useful synthesis of data for *N. sagittifera*. Additionally, the inclusion of this detailed account provides an important baseline for future work on cobra diversity in Myanmar, and we hope this will stimulate continued work on the fauna of the Coco Islands.

Figure 1: I recommend reorganising the country names, in order to avoid them not fitting in one line and/or getting confused with country borders. Adjusting or reducing the font size should be sufficient.

We have adjusted the country names as suggested.

Lines 181–184 (Caption to Figure 1): I suggest changing N. sp. to Naja sp. (also in the figure legend). Furthermore, the line drawing of N. kaouthia seems superfluous, and does not convey any relevant information.

Changes made as suggested

Line 188: I suggest changing "Species of Known Occurrence" to something like "Historically Documented Species".

Changed as suggested.

Line 227: Although traditionally considered a non-spitting cobra, current evidence indicates that N. kaouthia does spit venom in some parts of its distribution. Exactly as stated in lines 229 and 230, the occurrence of venom-spitting behaviour in this species in Myanmar cannot be ruled out a priori.

Please see our response to your comment above regarding Line 102.

Line 233: It is worth including Rahman et al., 2025 (https://doi.org/10.1016/j.toxicon.2024.108221) among the references regarding venom ophthalmia caused by N. kaouthia venom.

We have added this reference.

Lines 233–235: Please reference the official WHO guidelines for the management of snakebites (https://www.who.int/publications/i/item/9789290225300; page 164) and Chu et al., 2010 (<https://doi.org/10.1016/j.toxicon.2010.02.023>).

We have added these references.

Lines 263–266: Correctly interpreting the methodological approach of Neto et al. on the proteomics of *N. mandalayensis* venom is admittedly challenging. It is my understanding that the information presented in the pie charts does not represent an actual quantification of the relative abundances of the toxins in *N. mandalayensis* venom, but rather reflects their diversity. I suggest that the authors consider rephrasing this section accordingly.

We agree that the work of Neto et al. is not the most straightforward to interpret in a comparative sense. Considering this, the indicated sentence is not based on the data from the pie charts, and we avoided making direct comparisons across the two species based on the findings of Neto et al. We only note in our manuscript that the two venoms differ compositionally (which is supported by Neto) and that *N. mandalayensis* has a higher relative abundance of cardiotoxic 3Ftx than *N. kaouthia* following Neto’s Figure 4, and the sentence below from Neto et al.:

“Noteworthy to mention is the high relative concentration of the cardiotoxins (3FTx) standing out for *N. mandalayensis* venom in comparison to *N.* *kaouthia*.”

Our paper does not dive deeply into fine scale venom differences, but we instead use this information to contextualize the following sentence where we indicate that BPI Cobra Antivenom has been shown to offer cross-neutralization against *N. mandalayensis.*

Lines 272–274: Unless this traditional remedy has been demonstrated to have any medical efficacy, I recommend that the authors clearly state that its use should be discouraged in favour of the standard snakebite treatment and management procedures endorsed by the World Health Organization.

We have modified this sentence as follows:
“A traditional remedy involves using the leaf juice of tamarind (Tamarindus indica), chewed by the affected person or bystanders and dripped into the envenomed eyes [18], though this practice should be discouraged in favour of the standard snakebite treatment and management procedures endorsed by the World Health Organization [42].”

Lines 321–323: It may be worth mentioning that *N. fuxi* was previously considered *N. kaouthia*, which is recognised as Category 1 throughout its entire range in Myanmar.

We have adjusted this section (starting line 397) as follows:
“Due to its recent description, *Naja* *fuxi* has not yet been assessed by the World Health Organization [32], but it is anticipated to qualify as a Category 1 or Category 2 medically important snake throughout its distribution. Prior to its description, *Naja* *fuxi* was considered part of *N. kaouthia*, which is classified as a Category 1 medically important species in Myanmar [32].”

Lines 328–331: A similar situation applies to *N. kaouthia*, within which *N. fuxi* was originally classified.

Yes, though there seems to be a pronounced geographic component to this. We (the authors) have collectively handled hundreds of wild SE Asian *N. kaouthia* (Myanmar, Thailand, Cambodia, Vietnam, Malaysia) and have never had a snake spit venom when provoked. We discuss this in greater detail in Balchan et al. 2025 (https://doi.org/10.11646/zootaxa.5717.4.2). The first author (NRB) has been spat at by several wild *N. fuxi* in Vietnam, and the descriptions from China and Thailand also describe spitting. More work is needed to understand that spatial dimensions of spitting in *N. fuxi*, but it is certainly possible from at least three localities!

Lines 420–422: As already mentioned, the WHO Snakebite Information and Data Platform actually already lists N. sumatrana (as well as N. siamensis) among the medically relevant snake species of Myanmar.

We have modified this section (line 510) to reflect this:
*“Naja* *sumatrana* is classified as a Category 2 medically important snake in Myanmar [6,32].”

Line 486: Given the scarcity of behavioural data for most of the Naja species discussed here, I recommend adopting this more cautious phrasing also when referring to the (putative) “non-spitters” of Myanmar.

We have adjusted this (line 585) as follows to reflect the scarcity of data:
“*Naja sagittifera* is considered to be a non-spitting cobra, thought behavioral data are lacking for this species.”

Lines 495 and 496: I suggest explicitly naming the species to which the authors are referring, in order to avoid potential confusion.

This section has been rewritten (starting line 593) with the species explicitly named to avoid any confusion.

Lines 496–500: In my view, this section requires critical reconsideration, as the interpretation presented has several substantial limitations. First, the authors compare opportunistic observations with records derived from active searches. As these two data sources are not methodologically comparable, this appears to represents a classic “apples and oranges” problem. Moreover, encounter frequency alone cannot be assumed to reflect snake activity, and it is not possible to disentangle true biological patterns from variation in detection probability without correcting for sampling effort. The authors also treat the Thailand dataset as a biological baseline, although it appears to be another human-mediated dataset. Interpreting this pattern as biologically representative is not justified without independent validation.

We thank the reviewer for this comment, and the following two. We feel that these comments have brought up several valuable concerns. We have rewritten this section (starting line 593) to address this comment and the following two comments. We have included some statistical analyses and tempered our statements as suggested. We believe these changes have improved this section and are thankful for these comments.

Lines 501–509: Have the authors considered the possibility that the high proportion of encounters reported near human habitation reflects observer distribution rather than cobra distribution? Most observations may have occurred near homes because most observers were located there, not because cobras are disproportionately associated with human habitation.

Please see response above.

Lines 510–516: Assuming that encounter frequency predicts snakebite incidence likely constitutes an inference. Furthermore, agricultural and forestry activities in Myanmar are seasonal. As most bites occur during agricultural or forest-related work, exposure risk is expected to vary seasonally. In this context, the seasonality of snakebite may reflect work cycles rather than cobra visibility near homes.

Please see response above.

Line 527: The World Health Organization lists Myanmar Pharmaceutical Factory as a domestic producer. Is this the same producer mentioned here?

We have clarified that these are synonymous (lines 645-647):
“Currently, only two antivenoms are produced and distributed by Burmese Pharmaceutical Industries (Myanmar Pharmaceutical Factory), the sole domestic producer [10].”

Line 535: Here are a couple more recent references worth considering: https://doi.org/10.1371/journal.pntd.0000569; <https://doi.org/10.1038/nrdp.2017.63>

We have added these references and adjusted the sentence (lines 654-657):

“Barriers such as logistical constraints, healthcare infrastructure limitations, and rural access issues can considerably delay or prevent the administration of antivenom, especially in countries with limited resources [62–64].”

Line 553: I encourage the authors to clarify what they mean by “less harmful” methods. The traditional remedies listed here still delay the application of effective treatments, potentially increasing the severity of envenomation symptoms.

We have adjusted this sentence for clarity (Lines 675-677):
“Less harmful (lower likelihood of additional infection or trauma) but still ineffective methods included applying a “black stone” to the wound, drinking holy water or coconut water, reciting prayers, or placing a dead chick on the bite site.”

Lines 608–612: These considerations should be highlighted also when mentioning the relevance of traditional remedies.

We have emphasized this by adding a sentence of this nature (Lines 682-683):
“The administration of antivenom in a medical facility remains the only efficacious treatment for individuals exhibiting venom-induced toxic effects [10,42,72,73,77]”

Line 650: Is Myanmar Pharmaceutical Factory an alternative name for the Burmese Pharmaceutical Industries mentioned before?

We have clarified this (lines 772-776):
“Antivenoms with indications for the species discussed here are only indicated for a single species, *Naja kaouthia*: QSMI Cobra and Neuro polyvalent Snake Antivenin (Queen Saovabha Memorial Institute, Bangkok, Thailand) and anti-NK antivenom (Myanmar Pharmaceutical Factory - MPF; formerly Burma Pharmaceutical Industry - BPI) [42,85].”

Lines 700 and 701: Fasciotomy is not only not recommended, but is also often unnecessary when appropriate antivenom is administered successfully.

We have edited the sentence to reflect this (lines 829-833):
“Invasive surgery (fasciotomy) due to concerns of compartmental hypertension (compartment syndrome), although frequently performed in Asian and Southeast Asian *Naja* spp. envenoming cases is not a recommended practice. Minimal delay to good medical support and proper administration of appropriate antivenom will usually mitigate the development of elevated intracompartmental pressure [42,66,67,73,76].”

Lines 706–708: I suggest mentioning *N. kaouthia* from Myanmar as a potential spitter.

*Naja kaouthia* is already mentioned in this section, but we have added *N. fuxi* (Lines 838-839):

“*N. mandalayensis* [33] and *N. kaouthia* [40] are recognized species with documented ocular exposures due to the spitting of venom. Two additional spitting cobra species ranging in Myanmar are *N. siamensis* [66,91], and *N. sumatrana* [55], and venom spitting behavior is also known from *N. fuxi* [20,21].”

Abstract and Conclusion: In light of the points raised above and the amendments required, these two sections should be revised.

We have adjusted both sections with consideration for the points raised above.
