# Supplementary material for: The cobras (genus Naja) of Myanmar: An updated species list with information on identification, distributions, and medical importance
Source: PLoS Negl Trop Dis. 2026 Jun 29;20(6):e0014445. doi: 10.1371/journal.pntd.0014445 (PMC13340773; doi:10.1371/journal.pntd.0014445)
Supplement: Attachment — Submitted filename: Naja manuscript comments.docx [file pntd.0014445.s005.docx]

This resubmission is substantially stronger and much closer to being acceptable on scientific-soundness grounds. The manuscript now clearly states what the study does (a synthesis of Myanmar *Naja* diversity and distributions using vetted citizen-science records, museum specimens, and published sources) and, importantly, it has improved in three places that previously raised concerns: (i) the record-collection workflow is now much more explicit (platforms searched, dates, search terms, record counts per taxon), (ii) the encounter/seasonality analysis is appropriately framed as *reported encounters* with clear recognition of reporting bias, and (iii) the antivenom discussion is more carefully linked to evidence and explicitly acknowledges uncertainty and testing gaps.

There are still a few revisions needed to ensure the results are accurately reported, that the evidence thresholds are applied consistently, and that public health implications are stated with the right level of certainty. These changes should be achievable without new fieldwork.

Essential work needed for a revision

1) Standardize evidence language (“confirmed” vs “probable/possible”) across the manuscript
The Methods provide detailed record counts by species, which is excellent. However, the manuscript still uses strong “confirm” language in places where the evidentiary basis is thin (e.g., very small numbers of records). To prevent overstatement:

- Add a short “evidence tier” definition (voucher specimen; diagnostic photograph with traceable provenance; credible photo record; unconfirmed/inferred).
- Apply those tiers consistently in the Abstract, Results/Discussion summary, Table 1, and Conclusions.
  This is mainly wording and presentation, but it matters because clinicians and non-specialists may interpret “confirmed” as equally strong across taxa.

2) Improve reproducibility/verification of georeferencing and uncertainty handling
You report that location information was confirmed, often by contacting observers, and you plot point data on a national map. To make the work repeatable and to prevent overinterpretation of point precision:

- State explicitly how localities were converted to coordinates (exact GPS vs town centroid vs administrative centroid vs manual lookup).
- Add a locality-precision field in the supplementary occurrence dataset (e.g., exact / approximate / broad).
- Clarify how uncertain localities were treated in mapping and whether any sensitive localities were generalized.

3) Strengthen the citizen-science QA/QC description (small but important additions)
The revised Methods are good, but a reader still needs two clarifications:

- The rule used to merge duplicates across platforms (what counts as the same encounter).
- How ambiguous IDs were handled (e.g., conservative assignment to *Naja* sp. when diagnostic traits not visible).
  These can be short sentences, but they will substantially improve transparency.

4) Seasonality section: keep the interpretation conservative and self-contained
The manuscript now does a good job of stating that reporting bias likely shapes the pattern. To keep this fully aligned with “what the data show”:

- Ensure all phrasing refers to *reported encounters* (not cobra activity) in the Results/Discussion and figure caption.
- Consider one small robustness statement (optional but helpful): whether the dry-season peak persists when restricting to records with high-confidence month and locality (if that filter is already available in your dataset). If not feasible, keep the interpretation explicitly descriptive.

5) Antivenom and medical claims: tighten wording where the evidence is indirect
The manuscript appropriately highlights that locally produced antivenom targets only one cobra species and that cross-neutralization can be limited across taxa. Where direct neutralization/clinical evidence exists, keep it specific; where it does not, use conditional language (“may be limited,” “remains untested,” “should be evaluated”).
A concise summary table/box would make this stronger without adding new experiments: *species present → antivenom currently produced → evidence status for cross-neutralization → priority research need*.

6) Data availability: ensure the Supporting Information truly enables replication
You state that all relevant data are within the paper and supporting files. For this to meet the expectation in practice:

- Ensure S1 Appendix includes the full occurrence list (with source type, date, locality, coordinates, and uncertainty/precision class), and not only mapped points.
- If any records are restricted (privacy/sensitive localities), explain what is withheld and provide a suitable alternative (e.g., generalized coordinates) to keep the dataset usable.
