# Supplementary material for: The cobras (genus Naja) of Myanmar: An updated species list with information on identification, distributions, and medical importance
Source: PLoS Negl Trop Dis. 2026 Jun 29;20(6):e0014445. doi: 10.1371/journal.pntd.0014445 (PMC13340773; doi:10.1371/journal.pntd.0014445)
Supplement: Attachment — Submitted filename: Response to Reviewers.docx [file pntd.0014445.s006.docx]

**Reviewers' comments:**

Reviewer's Responses to Questions

**Key Review Criteria Required for Acceptance?**

As you describe the new analyses required for acceptance, please consider the following:

**Methods**


-Are the objectives of the study clearly articulated with a clear testable hypothesis stated?

-Is the study design appropriate to address the stated objectives?

-Is the population clearly described and appropriate for the hypothesis being tested?

-Is the sample size sufficient to ensure adequate power to address the hypothesis being tested?

-Were correct statistical analysis used to support conclusions?

-Are there concerns about ethical or regulatory requirements being met?

Reviewer #2: The authors have successfully addressed my comments regarding the methods they applied, improving their description in the text and acknowledging the limitations.

We thank the reviewer for their comments.

Reviewer #3: Objectives/hypothesis: The objectives are clear for a synthesis paper, but explicit “testable” claims would benefit from being stated as evidence-tiered conclusions (confirmed/probable/possible occurrences).
Study design: Appropriate (multi-source evidence synthesis + descriptive analyses).
Population described: The “population” is occurrence records; it is described reasonably well, but georeferencing precision and uncertainty treatment need clearer reporting.
Sample size/power: Adequate for the two common species used in encounter analysis; limited for taxa with very few records, requiring cautious inference.
Statistics: The chi-square framework for encounter timing is appropriate for reported encounter counts, provided interpretation remains descriptive and bias-aware.
Ethics/regulatory: No human-subject research is apparent. Ensure permissions/crediting for photographs and privacy/sensitive locality handling are documented.

We thank the review for these comments and have addressed the indicated points.

**Results**


-Does the analysis presented match the analysis plan?

-Are the results clearly and completely presented?

-Are the figures (Tables, Images) of sufficient quality for clarity?

Reviewer #2: The results appropriately match the analysis plan and are presented adequately.

We thank the reviewer for their comments.

Reviewer #3: The analyses reported generally match the described plan and are clearer than before. To be fully complete and verifiable, results should explicitly link each “confirmed/probable/possible” taxon claim to an evidence tier, and figures/tables should include (or point to) confidence/precision categories for records. Figures appear appropriate and interpretable, but map legends and any encounter figures should remain clearly labeled as reported encounters and remain readable at journal scale.

We have made these adjustments, modifying line 166: “We provide accounts for six *Naja* species with confirmed (voucher specimens or diagnostic photograph with traceable provenance) or potential (unconfirmed/inferred) occurrence in Myanmar: *Naja fuxi, N. kaouthia, N. mandalayensis, N. sagittifera, N. siamensis, and N. sumatrana*.”

**Conclusions**


-Are the conclusions supported by the data presented?

-Are the limitations of analysis clearly described?

-Do the authors discuss how these data can be helpful to advance our understanding of the topic under study?

-Is public health relevance addressed?

Reviewer #2: The authors have greatly improved the quality of the conclusions.

We thank the reviewer for their comments.

Reviewer #3: The conclusions are broadly supported, but must be calibrated by evidence strength (especially for taxa with few records and for antivenom inference). Limitations are now stated more clearly than before; still, they should be consolidated and placed where they directly constrain interpretation (especially for seasonality and public health statements). The paper is helpful for advancing understanding by updating national cobra diversity/distribution and highlighting antivenom testing needs. Public health relevance is clearly addressed, but needs careful conditional wording where efficacy evidence is indirect or absent.
We have modified these sections in accordance with specific comments in the included file and believe that these changes have improved the revised manuscript.

**Editorial and Data Presentation Modifications?**


Use this section for editorial suggestions as well as relatively minor modifications of existing data that would enhance clarity. If the only modifications needed are minor and/or editorial, you may wish to recommend “Minor Revision” or “Accept”.

Reviewer #2: Two minor comments:

1) The authors refer to the markings on the cobras’ hoods as “hood mark”, “hoodmark”, and “hood markings”. For the sake of consistency, I suggest using a single term throughout the text.

We thank the reviewer for bringing this to our attention, and we have changed wording to “hood mark(s)” in all instances within the manuscript for consistency.

2) I would like the authors to ensure that the scientific name of each species is written in full at least the first time it is mentioned in the manuscript.

We thank the reviewer for bringing this to our attention, and we have edited the introduction such that names are written out in entirety upon first mention for all species.

Reviewer #3: Minor-to-moderate edits would improve clarity: standardize “confirmed/probable/possible” terminology, add a short evidence-tier definition, include a locality-precision field in the dataset, and consider a brief antivenom-evidence summary table/box. Ensure all figures/tables remain legible and self-contained at publication size.

We have modified the methods and appendix accordingly. We have considered adding a summary table but have ultimately refrained from doing so as we feel that it would not provide the same perspective conveyed by the text.

**Summary and General Comments**


Use this section to provide overall comments, discuss strengths/weaknesses of the study, novelty, significance, general execution and scholarship. You may also include additional comments for the author, including concerns about dual publication, research ethics, or publication ethics. If requesting major revision, please articulate the new experiments that are needed.

Reviewer #2: I commend the authors for properly addressing my comments on the original version of the manuscript. I believe this revised version is suitable for publication in PLOS Neglected Tropical Diseases.

We thank the reviewer for their constructive comments.

Reviewer #3: This is a valuable synthesis with clear relevance to snakebite management. The manuscript is much improved and close to acceptable. Remaining weaknesses are largely about transparency (georeferencing uncertainty) and ensuring that strong claims are reserved for strong evidence. No new experiments are essential; targeted reporting and wording revisions should be sufficient.

We thank the reviewer for their constructive comments and believe that they have improved our revised manuscript.

This resubmission is substantially stronger and much closer to being acceptable on scientific-soundness grounds. The manuscript now clearly states what the study does (a synthesis of Myanmar *Naja* diversity and distributions using vetted citizen-science records, museum specimens, and published sources) and, importantly, it has improved in three places that previously raised concerns: (i) the record-collection workflow is now much more explicit (platforms searched, dates, search terms, record counts per taxon), (ii) the encounter/seasonality analysis is appropriately framed as *reported encounters* with clear recognition of reporting bias, and (iii) the antivenom discussion is more carefully linked to evidence and explicitly acknowledges uncertainty and testing gaps.

We thank the reviewer for this positive assessment of our revised manuscript.

There are still a few revisions needed to ensure the results are accurately reported, that the evidence thresholds are applied consistently, and that public health implications are stated with the right level of certainty. These changes should be achievable without new fieldwork.

We have addressed each of these comments individually as follows below.

Essential work needed for a revision

1) Standardize evidence language (“confirmed” vs “probable/possible”) across the manuscript
The Methods provide detailed record counts by species, which is excellent. However, the manuscript still uses strong “confirm” language in places where the evidentiary basis is thin (e.g., very small numbers of records). To prevent overstatement:

- Add a short “evidence tier” definition (voucher specimen; diagnostic photograph with traceable provenance; credible photo record; unconfirmed/inferred).
- Apply those tiers consistently in the Abstract, Results/Discussion summary, Table 1, and Conclusions.
  This is mainly wording and presentation, but it matters because clinicians and non-specialists may interpret “confirmed” as equally strong across taxa.

We thank the reviewer for this comment and agree that this additional clarity is valuable. Accordingly, we have modified the methods at line 166: “We provide accounts for six *Naja* species with confirmed (voucher specimens or diagnostic photograph with traceable provenance) or potential (unconfirmed/inferred) occurrence in Myanmar: *Naja fuxi, N. kaouthia, N. mandalayensis, N. sagittifera, N. siamensis, and N. sumatrana*.”

We have also modified wording in the Abstract, Results/Discussion, Table 1 caption, and Conclusions to reflect this standardized language and improve clarity.

2) Improve reproducibility/verification of georeferencing and uncertainty handling
You report that location information was confirmed, often by contacting observers, and you plot point data on a national map. To make the work repeatable and to prevent overinterpretation of point precision:

- State explicitly how localities were converted to coordinates (exact GPS vs town centroid vs administrative centroid vs manual lookup).
  We have added this (line 143): “Coordinates for all citizen science records were obtained through manual georeferencing of locality data using Google Earth, and no locality points were further obscured or generalized.”
- Add a locality-precision field in the supplementary occurrence dataset (e.g., exact / approximate / broad).
  We have added a locality precision field to the supplementary dataset with “exact” and “approximate”.
- Clarify how uncertain localities were treated in mapping and whether any sensitive localities were generalized.
  Please see comment above regarding line 144.

3) Strengthen the citizen-science QA/QC description (small but important additions)
The revised Methods are good, but a reader still needs two clarifications:

- The rule used to merge duplicates across platforms (what counts as the same encounter).
- How ambiguous IDs were handled (e.g., conservative assignment to *Naja* sp. when diagnostic traits not visible).
  These can be short sentences, but they will substantially improve transparency.

  We thank the reviewer for raising these points, and have modified this sentence (line 181) accordingly: “Duplicate records (e.g., the same image posted to both Facebook and iNaturalist) were combined to avoid overcounting individual encounters, voucher specimens were omitted to avoid biases associated with intentionally collected snakes, and ambiguous records (identified as *Naja* sp.) were not included.”

4) Seasonality section: keep the interpretation conservative and self-contained
The manuscript now does a good job of stating that reporting bias likely shapes the pattern. To keep this fully aligned with “what the data show”:

- Ensure all phrasing refers to *reported encounters* (not cobra activity) in the Results/Discussion and figure caption.
  We thank the reviewer for raising this point, and we have adjusted the wording in several areas throughout the manuscript to reflect “reported encounters”.
- Consider one small robustness statement (optional but helpful): whether the dry-season peak persists when restricting to records with high-confidence month and locality (if that filter is already available in your dataset). If not feasible, keep the interpretation explicitly descriptive.
  The month and locality data are high confidence for all records used in this analysis, so this will be infeasible for our dataset. We have maintained an explicitly descriptive narrative in the discussion associated with dry season peaks.

5) Antivenom and medical claims: tighten wording where the evidence is indirect
The manuscript appropriately highlights that locally produced antivenom targets only one cobra species and that cross-neutralization can be limited across taxa. Where direct neutralization/clinical evidence exists, keep it specific; where it does not, use conditional language (“may be limited,” “remains untested,” “should be evaluated”).
A concise summary table/box would make this stronger without adding new experiments: *species present → antivenom currently produced → evidence status for cross-neutralization → priority research need*.

We thank the reviewer for these comments, and we have rewritten a large portion of this discussion (lines 739-795). We have considered adding a summary table but have ultimately refrained from doing so as we feel that it would not provide the same perspective conveyed by the text.

6) Data availability: ensure the Supporting Information truly enables replication
You state that all relevant data are within the paper and supporting files. For this to meet the expectation in practice:

- Ensure S1 Appendix includes the full occurrence list (with source type, date, locality, coordinates, and uncertainty/precision class), and not only mapped points.
  We thank the reviewer for raising these points and we have modified the Appendix file to include fields for “source type” and “locality precision”.
- If any records are restricted (privacy/sensitive localities), explain what is withheld and provide a suitable alternative (e.g., generalized coordinates) to keep the dataset usable.
  No records are restricted in this dataset, and we have added a “locality precision” field to the supplemental file.
