# Supplementary material for: The cobras (genus Naja) of Myanmar: An updated species list with information on identification, distributions, and medical importance
Source: PLoS Negl Trop Dis. 2026 Jun 29;20(6):e0014445. doi: 10.1371/journal.pntd.0014445 (PMC13340773; doi:10.1371/journal.pntd.0014445)
Supplement: Peer review history

# Peer review: Decision Letter 0

**DOI:** 10.1371/journal.pntd.0014445.r001

**Authors:** Wuelton Monteiro

19 Feb 2026

PNTD-D-26-00158

The cobras (genus *Naja* ) of Myanmar: An updated species list with information on identification, distributions, and medical importance

PLOS Neglected Tropical Diseases

Dear Dr. Wüster,

Thank you for submitting your manuscript to PLOS Neglected Tropical Diseases. After careful consideration, we feel that it has merit but does not fully meet PLOS Neglected Tropical Diseases's publication criteria as it currently stands. Therefore, we invite you to submit a revised version of the manuscript that addresses the points raised during the review process.

Please submit your revised manuscript within by Apr 20 2026 11:59PM. If you will need more time than this to complete your revisions, please reply to this message or contact the journal office at plosntds@plos.org. When you're ready to submit your revision, log on to https://www.editorialmanager.com/pntd/ and select the 'Submissions Needing Revision' folder to locate your manuscript file.

Please include the following items when submitting your revised manuscript:

* A letter that responds to each point raised by the editor and reviewer(s). You should upload this letter as a separate file labeled 'Response to Reviewers'. This file does not need to include responses to any formatting updates and technical items listed in the 'Journal Requirements' section below.

* A marked-up copy of your manuscript that highlights changes made to the original version. You should upload this as a separate file labeled 'Revised Manuscript with Track Changes'.

* An unmarked version of your revised paper without tracked changes. You should upload this as a separate file labeled 'Manuscript'.

If you would like to make changes to your financial disclosure, competing interests statement, or data availability statement, please make these updates within the submission form at the time of resubmission. Guidelines for resubmitting your figure files are available below the reviewer comments at the end of this letter.

We look forward to receiving your revised manuscript.

Kind regards,

Wuelton Monteiro, Ph.D.

Section Editor

PLOS Neglected Tropical Diseases

Wuelton Monteiro

Section Editor

PLOS Neglected Tropical Diseases

Shaden Kamhawi

co-Editor-in-Chief

PLOS Neglected Tropical Diseases

orcid.org/0000-0003-4304-636XX

Paul Brindley

co-Editor-in-Chief

PLOS Neglected Tropical Diseases

orcid.org/0000-0003-1765-0002

**Journal Requirements:**

1) We ask that a manuscript source file is provided at Revision. Please upload your manuscript file as a .doc, .docx, .rtf or .tex. If you are providing a .tex file, please upload it under the item type u2018LaTeX Source Fileu2019 and leave your .pdf version as the item type u2018Manuscriptu2019.

2) Please upload all main figures as separate Figure files in .tif or .eps format. For more information about how to convert and format your figure files please see our guidelines:

https://journals.plos.org/plosntds/s/figures

3) Some material included in your submission may be copyrighted. According to PLOSu2019s copyright policy, authors who use figures or other material (e.g., graphics, clipart, maps) from another author or copyright holder must demonstrate or obtain permission to publish this material under the Creative Commons Attribution 4.0 International (CC BY 4.0) License used by PLOS journals. Please closely review the details of PLOSu2019s copyright requirements here: PLOS Licenses and Copyright. If you need to request permissions from a copyright holder, you may use PLOS's Copyright Content Permission form.

Please respond directly to this email and provide any known details concerning your material's license terms and permissions required for reuse, even if you have not yet obtained copyright permissions or are unsure of your material's copyright compatibility. Once you have responded and addressed all other outstanding technical requirements, you may resubmit your manuscript within Editorial Manager.

Potential Copyright Issues:

- Please confirm (a) that you are the photographer of Figures 2, 3, 4, 5, 6, & 7:  or (b) provide written permission from the photographer to publish the photo(s) under our CC BY 4.0 license.

- Figure 1: Please (a) provide a direct link to the base layer of the map (i.e., the country or region border shape) and ensure this is also included in the figure legend; and (b) provide a link to the terms of use / license information for the base layer image or shapefile. We cannot publish proprietary or copyrighted maps (e.g. Google Maps, Mapquest) and the terms of use for your map base layer must be compatible with our CC BY 4.0 license.

Note: if you created the map in a software program like R or ArcGIS, please locate and indicate the source of the basemap shapefile onto which data has been plotted.

If your map was obtained from a copyrighted source please amend the figure so that the base map used is from an openly available source. Alternatively, please provide explicit written permission from the copyright holder granting you the right to publish the material under our CC BY 4.0 license.

If you are unsure whether you can use a map or not, please do reach out and we will be able to help you. The following websites are good examples of where you can source open access or public domain maps:

* U.S. Geological Survey (USGS) - All maps are in the public domain. (http://www.usgs.gov)

* PlaniGlobe - All maps are published under a Creative Commons license so please cite u201cPlaniGlobe, http://www.planiglobe.com, CC BY 2.0u201d in the image credit after the caption. (http://www.planiglobe.com/?lang=enl)

* Natural Earth - All maps are public domain. (http://www.naturalearthdata.com/about/terms-of-use/).

**Reviewers' Comments:**

Reviewer's Responses to Questions

**Key Review Criteria Required for Acceptance?**

As you describe the new analyses required for acceptance, please consider the following:

**Methods**

-Are the objectives of the study clearly articulated with a clear testable hypothesis stated?

-Is the study design appropriate to address the stated objectives?

-Is the population clearly described and appropriate for the hypothesis being tested?

-Is the sample size sufficient to ensure adequate power to address the hypothesis being tested?

-Were correct statistical analysis used to support conclusions?

-Are there concerns about ethical or regulatory requirements being met?

Reviewer #1: The methods are well described and adequate for the type of study. Using citizen science data is a useful approach for filling knowledge gaps in crisis disciplines, such as snakebite mitigation, where additional data is urgently needed but difficult to obtain through more conventional data collection approaches. Any limitations associated with this approach are clearly discussed in the manuscript.

Reviewer #2: Details provided in the attached PDF file.

Reviewer #3: This manuscript aims to clarify which Naja cobra species occur in Myanmar, update their distributions using a synthesis of museum/literature evidence and vetted photographic (citizen-science/social media) records, and discuss implications for snakebite management. The objectives are generally clear for a synthesis paper, but the manuscript would benefit from sharper, explicitly stated, testable claims that align with the evidence assembled—e.g., “Species X occurs in Myanmar,” “Species Y is restricted to region Z,” or “reported encounters peak in months A–B.” As written, some statements read as definitive confirmation even where the underlying evidence appears sparse. For acceptance, the manuscript should consistently differentiate what is directly demonstrated by vouchered specimens or clearly diagnostic, traceable photographic records from what is probable or possible based on limited or lower-confidence reports. This is essential to ensure the conclusions are accurately reported and scaled to the strength of evidence.

The overall study design is appropriate for the stated aims. A mixed-source occurrence synthesis is a reasonable approach in settings where systematic survey coverage is limited. However, to meet acceptance criteria, the design must be reported with sufficient transparency that another team could replicate the record-assembly and vetting workflow and reach comparable inferences. In particular, the manuscript should explicitly describe the universe of sources searched (platforms, museum collections, and literature scope), the time window covered, inclusion/exclusion rules, how duplicates were handled, how locality information was georeferenced (and how uncertain localities were treated), and how identification decisions were made and adjudicated when ambiguous. Because citizen-science/social-media observations are inherently biased toward accessible locations, settlements, and user participation, the Methods should explicitly acknowledge this bias and the Results/Discussion should interpret “seasonality” as reported encounter seasonality rather than true cobra activity seasonality unless independent evidence supports biological inference.

In this work, the relevant “population” is the set of cobra occurrence records in Myanmar, not human participants. That population needs clearer operational definition—what constitutes a Myanmar record when locality precision varies, and what precision thresholds are required for inclusion in distribution mapping. The adequacy of sample size is uneven across taxa: where taxa are supported by multiple independent records and/or vouchers, descriptive distribution updates are plausible. Where taxa are supported by very few records (possibly a single record), this is still valuable, but it cannot sustain strong claims about broad distribution, stable seasonal patterns, or downstream clinical implications without careful qualification. Acceptance therefore depends on aligning the strength of statements with record volume and confidence: sparse-record taxa should be framed as probable/possible unless supported by high-tier evidence, and any temporal inference should be restricted to records with reliable date metadata.

The statistical components appear largely descriptive, which is appropriate for a synthesis paper, but the key issue is interpretive validity rather than choice of tests. Any seasonal patterns derived from opportunistic reporting should be presented cautiously and accompanied by either a simple robustness check or clearly stated limitations. For acceptance, the authors should either (i) provide a minimal sensitivity analysis repeating the seasonal plot using only high-confidence records with precise dates and locations and stating whether the pattern persists, or (ii) substantially rewrite the seasonality section so it remains descriptive and does not imply ecological causation. Similarly, medical/antivenom statements require careful calibration: any claim about antivenom efficacy or cross-neutralization must be tied explicitly to the type of evidence available (clinical vs preclinical vs inference). Where direct evidence is absent, the manuscript should use conditional language and frame recommendations as priorities for evaluation rather than implied proven adequacy or inadequacy.

Ethical and regulatory considerations are limited because there is no direct human-subject involvement. However, publication ethics transparency is still essential because the manuscript uses social-media images and in some cases observer communications. For acceptance, the manuscript should include a concise ethics/permissions statement covering: permissions and crediting for photographs used in figures, anonymization (no personally identifying information disclosed), and how sensitive locality information is handled (e.g., avoiding unnecessary precision where it could create risk). These are typically straightforward additions but important for compliance and reader trust.

In practical terms, the essential work required for acceptance is achievable within a short revision cycle and does not require new field collections. The authors should implement a clear evidence-tier framework (voucher/diagnostic photo/probable/possible) and apply it consistently across tables, species accounts, abstract, and conclusions; expand Methods to make the citizen-science workflow reproducible (sources searched, search strategy, screening criteria, duplicate rules, georeferencing, identification adjudication); add uncertainty flags to the supplementary dataset (ID confidence, locality precision, record type, diagnostic visibility); address seasonality interpretation through either a simple robustness check or a rewrite that emphasizes reporting bias; tighten all antivenom/medical claims so they strictly reflect available evidence; and include an explicit ethics/permissions statement for social-media content. With these targeted revisions, the manuscript would more reliably communicate what the data show, transparently convey limitations, and provide a reproducible, conservative update of Myanmar’s Naja cobra composition and distribution with appropriately framed public-health implications.

Reviewer #4: No comments or concerns.

**********

**Results**

-Does the analysis presented match the analysis plan?

-Are the results clearly and completely presented?

-Are the figures (Tables, Images) of sufficient quality for clarity?

Reviewer #1: The results appropriately summarize the collected data and are clearly presented.

Reviewer #2: Details provided in the attached PDF file.

Reviewer #3: The Results section is broadly aligned with the manuscript’s intended workflow—assembling and vetting occurrence records from multiple sources and presenting the outcomes as tables, maps, and descriptive summaries. However, acceptance will depend on making the Results more transparently traceable to the analysis plan, more complete in reporting the evidence base behind each conclusion, and clearer and more publication-ready in its figures and tables.

The findings reflect a compilation-and-synthesis approach consistent with the stated aims. Where the match is currently weakest is in demonstrating the outputs of key curation steps that are implied in the Methods (screening, deduplication, georeferencing, identification adjudication, and confidence assessment). To show clear alignment with the analysis plan, the Results should report at least minimal summary counts—how many records were identified, how many removed as duplicates, how many excluded for insufficient diagnostic quality or uncertain locality/date, and how many retained per species and per record type. Without these counts, it is difficult for readers to evaluate whether the presented distributions and species status statements arise from a structured analysis plan or from an unquantified selection process.

The findings are also generally understandable, but they are not yet complete in the sense that a reader can verify the strength of evidence behind each major claim. For acceptance, the Results should consistently quantify the evidence supporting each species’ status and distribution: number of retained records per species, broken down by record type (voucher specimen, diagnostic photograph, literature-only), and—if you adopt the recommended approach—by an explicit confidence/evidence tier and locality precision category. This is especially important for taxa supported by very few records; those results remain valuable, but the narrative must clearly reflect the limited sample size and should avoid definitive phrasing unless supported by high-tier evidence. In addition, temporal/seasonality results should be framed explicitly as reported encounter patterns and should present sample sizes so that readers can judge robustness; if feasible, a brief sensitivity statement using high-confidence records only would further strengthen completeness and prevent overinterpretation.

The figures and tables are central to this manuscript, but several need improvement to meet publication standards for clarity and self-contained interpretation. Key tables should define evidence categories and abbreviations in footnotes and include record counts per species by type and confidence. Maps should use consistent symbology across panels, include clear legends, and remain readable at journal print size; they should also clarify what locality precision is shown and caution against interpreting point density as abundance. Photographic figures (if included) should show diagnostic traits clearly, include proper credits/permissions, avoid revealing sensitive information, and use captions that state what the image demonstrates diagnostically. Seasonal and summary plots should clearly label axes/units, include sample sizes, and avoid visual designs that imply causation (e.g., rainfall overlays) unless the text and caption explicitly treat them as descriptive comparisons.

For the results to be acceptable, the authors should: (1) add a consolidated Results table listing, for each species, the total number of retained records and the breakdown by voucher/diagnostic photo/literature-only (and ideally by confidence tier and locality precision); (2) include a short screening/curation summary (records found → duplicates removed → excluded → retained) to demonstrate the Results match the analysis plan; (3) rewrite Results statements so that “confirmed/probable/possible” wording strictly matches the evidence tier and record volume; (4) treat seasonality explicitly as reported encounters, report sample sizes, and either add a simple high-confidence sensitivity note or keep interpretation strictly descriptive; and (5) strengthen captions so every figure/table is standalone, legible at publication size, and clear about what can and cannot be concluded. These changes do not require new fieldwork but are essential to ensure the Results are accurately reported and interpretable.

Reviewer #4: Overall the results are clearly presented. Some figures lack appropriate photographer credit within the captions, and this should be updated.

**********

**Conclusions**

-Are the conclusions supported by the data presented?

-Are the limitations of analysis clearly described?

-Do the authors discuss how these data can be helpful to advance our understanding of the topic under study?

-Is public health relevance addressed?

Reviewer #1: The conclusions correctly reflect the results, are well presented, and address any limitations of the study. My only concern would be that the elaborate discussion of snakebite management is not directly related to the collected data but is more of a review. However, I agree that the inclusion of these sections is a very useful addition because it makes all information on the topic of cobras and their bites in Myanmar easily accessible for readers in one manuscript.

Reviewer #2: Details provided in the attached PDF file.

Reviewer #3: The conclusions are broadly consistent with the manuscript’s purpose—providing an updated synthesis of Naja species occurrence and distribution in Myanmar with implications for snakebite management—but several statements need to be recalibrated so they strictly reflect what the compiled evidence supports. For acceptance, definitive wording such as “confirmed presence,” strong distribution claims, or statements implying robust seasonality should be reserved for taxa and patterns supported by high-confidence evidence (vouchered specimens or clearly diagnostic, traceable records). Where evidence is sparse or opportunistic, conclusions should be phrased as “supported by limited records,” “probable,” or “requiring confirmation,” and temporal patterns should be described as reported encounter patterns rather than biological seasonality unless independently validated.

The limitations are not yet stated clearly enough in the concluding section. A short, explicit limitations paragraph should be added immediately before (or within) the Conclusions to constrain interpretation. This should include: strong reporting/sampling bias in citizen-science sources, uneven geographic coverage, variable locality/date precision, possible misidentification from non-diagnostic images, and small sample sizes for some taxa. Where the manuscript draws medical implications, it should also plainly note limits to inference about antivenom performance (e.g., geographically structured venom variation and limited direct neutralization/clinical evidence for some taxa).

The manuscript can advance understanding in practical and defensible ways, and the Conclusions should state these more concretely: it provides a clearer, evidence-based national picture of cobra species composition and distribution; it identifies priority geographic and taxonomic gaps for targeted field surveys and voucher-based confirmation; and it offers a rational basis for prioritizing venom sampling and antivenom cross-neutralization testing.

Public health relevance is appropriately raised, but because this is high-stakes, the closing statements should be framed cautiously and operationally. Rather than implying proven antivenom efficacy or failure where direct evidence is not presented, the Conclusions should emphasize what the data allow: improved clarity on likely offending species distributions, better targeting of identification and training resources, and explicit priorities for evaluating antivenom coverage and strengthening surveillance. With these targeted revisions, the Conclusions will be evidence-aligned, transparent about uncertainty, and more useful for both biodiversity and snakebite stakeholders.

Reviewer #4: No comments or corrections. The data and results presented are of significant public health relevance.

**********

**Editorial and Data Presentation Modifications?**

Use this section for editorial suggestions as well as relatively minor modifications of existing data that would enhance clarity. If the only modifications needed are minor and/or editorial, you may wish to recommend “Minor Revision” or “Accept”.

Reviewer #1: L 428: reference (Leong et al., 2015) is not formatted correctly,

Reviewer #2: Details provided in the attached PDF file.

Reviewer #3: The manuscript would benefit from several editorial and data-presentation refinements that do not require new data collection but would substantially improve clarity, traceability, and reader confidence.

First, improve consistency and precision of terminology throughout. Use one standard set of terms for records (e.g., “voucher specimen,” “diagnostic photograph,” “literature record,” “unverified report”) and apply them consistently in the text, tables, and captions. Avoid mixing “confirmed/verified/recorded” interchangeably unless you define each term and use it systematically. Where you discuss temporal patterns, use “reported encounters/observations” rather than “activity” unless you explicitly justify biological inference.

Second, strengthen figure and table self-containment. Key tables should include clear footnotes defining abbreviations, evidence categories, and any confidence classes. Where you summarise species presence and distribution, include basic counts (number of retained records by type and confidence tier) directly in the main table rather than leaving these details implicit. Even if you already have these in supplementary files, a compact main-table version makes the paper much easier to interpret.

Third, improve map readability and comparability. Ensure consistent symbology across panels, legible administrative labels at journal print size, and standardized legends. If multiple maps are presented, standardize scale bars and north arrows. Consider using distinct symbols for record types (e.g., voucher vs photo) and, if you include uncertainty, a simple visual cue for locality precision (exact vs approximate). Add a short note in the caption cautioning that point density reflects reporting effort as well as occurrence.

Fourth, refine photographic figure presentation (if included). Ensure all photos used in figures have explicit credits and permission statements as required, highlight diagnostic features (e.g., with unobtrusive arrows or inset zooms if allowed by the journal), and avoid any content that reveals sensitive personal information or overly precise sensitive localities.

Fifth, tidy Results/Discussion structure to reduce repetition and improve flow. Use consistent subheadings that separate (i) species occurrence/distribution updates, (ii) encounter timing/seasonality, and (iii) medical/public health implications. Keep Results descriptive and move mechanistic explanations into Discussion to avoid over-interpretation.

Sixth, ensure the supplementary dataset is clean and maximally useful. Include standardized fields such as record source, record type, date, locality description, coordinates, coordinate uncertainty/precision class, identification confidence, and whether a voucher/photo exists. Check for duplicate rows, inconsistent place naming, and missing units. A brief “data dictionary” paragraph (or table) in the supplement would further improve usability.

Reviewer #4: Discussed in summary.

**********

**Summary and General Comments**

Use this section to provide overall comments, discuss strengths/weaknesses of the study, novelty, significance, general execution and scholarship. You may also include additional comments for the author, including concerns about dual publication, research ethics, or publication ethics. If requesting major revision, please articulate the new experiments that are needed.

Reviewer #1: The manuscript thoroughly reviews available data on cobra bite management in Myanmar and analyses important new data on cobra distributions and available antivenoms. Venomous snake distributions are often insufficiently understood and clarifying them is crucial for adequate snakebite management, since snakes are often not identifiable by victims and treatment needs to be decided on based on which snakes are present in the region.

Reviewer #2: Details provided in the attached PDF file.

Reviewer #3: This manuscript provides a timely synthesis of Myanmar’s Naja cobras by combining multiple evidence streams (museum/literature sources and vetted photographic/citizen-science records) to update species composition, distribution patterns, and the likely implications for snakebite management. The main strength is its practical intent: clarifying which cobra taxa are present and where they occur is valuable for clinicians, public health planning, and biodiversity monitoring. The work also demonstrates strong scholarship in assembling dispersed information into a single narrative and presenting it in a way that can be operationally useful.

The main weakness is not the idea, but the calibration of claims to evidence strength. Some summary statements read as definitive confirmations even where the underlying record base appears sparse or derived from opportunistic reporting with variable metadata quality. This matters because the paper’s conclusions are likely to be used beyond herpetology (e.g., in clinical contexts), where overstated certainty can mislead. A second weakness is reproducibility: the record-vetting and georeferencing workflow is described in general terms, but key details are missing (search strategy, inclusion/exclusion rules, duplicate handling, confidence assignment, and identification adjudication). Without these, readers cannot fully evaluate bias and cannot replicate the dataset assembly.

Novelty is moderate but meaningful for the target context: the manuscript’s value lies less in a new analytical method and more in an updated, evidence-informed national synthesis that integrates underused citizen-science observations with curated records. The public health significance is potentially high, but only if the medical statements are framed conservatively and explicitly tied to the type of evidence available (clinical vs preclinical vs inference), and if limitations (reporting bias, uneven coverage, locality/date uncertainty, and sparse records for some taxa) are clearly stated and used to bound conclusions.

No clear dual-publication concerns are apparent from the manuscript as reviewed, but publication ethics requirements should be handled explicitly given the use of social-media photographs: permissions/crediting, observer privacy/anonymization, and handling of sensitive locality information should be clearly stated. There are no apparent ethical concerns involving human participants, but the paper should confirm that any private communications were used with consent and that personal identifiers are not disclosed.

The essential “information requested does not need to be new experiments in the laboratory or new field collections. Rather, it should be limited, feasible, and focused on ensuring the results are accurately reported and reproducible: (1) implement an explicit evidence-tier framework (voucher/diagnostic photo/probable/possible) and apply it consistently across the abstract, tables, species accounts, and conclusions; (2) provide a fully reproducible description of the citizen-science record acquisition and vetting workflow (platforms searched, search terms, date range, inclusion/exclusion criteria, duplicate rules, georeferencing method, and ID adjudication); (3) include confidence and locality-precision flags in the supplementary occurrence dataset; and (4) either add a simple robustness check for the seasonality pattern using high-confidence records or rewrite that section to remain strictly descriptive of reported encounters. These revisions should be achievable within a short revision cycle and would substantially strengthen credibility, interpretability, and public health utility.

Reviewer #4: In this manuscript Balchan and colleagues review and update the current knowledge on the distribution, identification, medical relevance and overall envonoming treatment of medically significant cobras (Naja spp) in Myanmar. I found the study to be quite thorough and informative, including relevant natural history and distributional data for all species. I have one minor suggestion that I think is relatively important to address, that will aid in the overall usefulness of this paper as a tool for cobra identification within Myanmar. Although the authors are experts on snake identification and they do explicitly discuss morphological characters used to distinguish among the species in question, often these characters overlap, and the important characters (such as hood markings) are not always shown in their figures illustrative of "typical" examples of the species within the region (Figs 2-6). My suggestion is that the authors update these figures to include 1) insets of hood markings (either illustration or photos) typically associated with each species, and 2) a description in the caption of exactly which characters were used to determine the IDs of the specific individuals in the photos. This way readers will have an easier grasp on how IDs were determined.

Otherwise I found the manuscript to be quite a fine and important addition to the literature of medically relevant snakes in this understudied region.

**********

PLOS authors have the option to publish the peer review history of their article (what does this mean?). If published, this will include your full peer review and any attached files.

If you choose “no”, your identity will remain anonymous but your review may still be made public.

**Do you want your identity to be public for this peer review?**  For information about this choice, including consent withdrawal, please see our Privacy Policy.

Reviewer #1: **Yes:** Anna Pintor

Reviewer #2: No

Reviewer #3: **Yes:** Wisdom M.D. Dlamini

Reviewer #4: No

[NOTE: If reviewer comments were submitted as an attachment file, they will be attached to this email and accessible via the submission site. Please log into your account, locate the manuscript record, and check for the action link "View Attachments". If this link does not appear, there are no attachment files.]

**Figure resubmission:** While revising your submission, we strongly recommend that you use PLOS’s NAAS tool (https://ngplosjournals.pagemajik.ai/artanalysis) to test your figure files. NAAS can convert your figure files to the TIFF file type and meet basic requirements (such as print size, resolution), or provide you with a report on issues that do not meet our requirements and that NAAS cannot fix.

After uploading your figures to PLOS’s NAAS tool - https://ngplosjournals.pagemajik.ai/artanalysis, NAAS will process the files provided and display the results in the "Uploaded Files" section of the page as the processing is complete. If the uploaded figures meet our requirements (or NAAS is able to fix the files to meet our requirements), the figure will be marked as "fixed" above. If NAAS is unable to fix the files, a red "failed" label will appear above. When NAAS has confirmed that the figure files meet our requirements, please download the file via the download option, and include these NAAS processed figure files when submitting your revised manuscript.

**Reproducibility:**

To enhance the reproducibility of your results, we recommend that authors of applicable studies deposit laboratory protocols in protocols.io, where a protocol can be assigned its own identifier (DOI) such that it can be cited independently in the future. Additionally, PLOS ONE offers an option to publish peer-reviewed clinical study protocols. Read more information on sharing protocols at https://plos.org/protocols?utm_medium=editorial-email&utm_source=authorletters&utm_campaign=protocols

Attachment Submitted filename: PLoS_NTD_reviewer.pdf

# Author comment: Author response to Decision Letter 1

**DOI:** 10.1371/journal.pntd.0014445.r002

23 Apr 2026

Attachment Submitted filename: Responses to Reviewers.docx

# Peer review: Decision Letter 1

**DOI:** 10.1371/journal.pntd.0014445.r003

**Authors:** Wuelton Monteiro

11 May 2026

PNTD-D-26-00158R1The cobras (genus *Naja* ) of Myanmar: An updated species list with information on identification, distributions, and medical importancePLOS Neglected Tropical Diseases  Dear Dr. Wüster, Thank you for submitting your manuscript to PLOS Neglected Tropical Diseases. After careful consideration, we feel that it has merit but does not fully meet PLOS Neglected Tropical Diseases's publication criteria as it currently stands. Therefore, we invite you to submit a revised version of the manuscript that addresses the points raised during the review process. Please submit your revised manuscript by Jun 10 2026 11:59PM. If you will need more time than this to complete your revisions, please reply to this message or contact the journal office at plosntds@plos.org. When you're ready to submit your revision, log on to https://www.editorialmanager.com/pntd/ and select the 'Submissions Needing Revision' folder to locate your manuscript file. Please include the following items when submitting your revised manuscript:* A letter that responds to each point raised by the editor and reviewer(s). You should upload this letter as a separate file labeled 'Response to Reviewers'. This file does not need to include responses to any formatting updates and technical items listed in the 'Journal Requirements' section below.* A marked-up copy of your manuscript that highlights changes made to the original version. You should upload this as a separate file labeled 'Revised Manuscript with Track Changes'.* An unmarked version of your revised paper without tracked changes. You should upload this as a separate file labeled 'Manuscript'. If you would like to make changes to your financial disclosure, competing interests statement, or data availability statement, please make these updates within the submission form at the time of resubmission. Guidelines for resubmitting your figure files are available below the reviewer comments at the end of this letter. As the corresponding author, your ORCID iD is verified in the submission system and will appear in the published article. PLOS supports the use of ORCID, and we encourage all coauthors to register for an ORCID iD and use it as well. Please encourage your coauthors to verify their ORCID iD within the submission system before final acceptance, as unverified ORCID iDs will not appear in the published article. Only the individual author can complete the verification step; PLOS staff cannot verify ORCID iDs on behalf of authors.We look forward to receiving your revised manuscript. Kind regards, Wuelton Monteiro, Ph.D.Section EditorPLOS Neglected Tropical Diseases Wuelton MonteiroSection EditorPLOS Neglected Tropical Diseases

Shaden Kamhawi

co-Editor-in-Chief

PLOS Neglected Tropical Diseases

orcid.org/0000-0003-4304-636XX

Paul Brindley

co-Editor-in-Chief

PLOS Neglected Tropical Diseases

orcid.org/0000-0003-1765-0002

**Reviewers' comments:**  Reviewer's Responses to Questions

**Key Review Criteria Required for Acceptance?**

As you describe the new analyses required for acceptance, please consider the following:

**Methods**

-Are the objectives of the study clearly articulated with a clear testable hypothesis stated?

-Is the study design appropriate to address the stated objectives?

-Is the population clearly described and appropriate for the hypothesis being tested?

-Is the sample size sufficient to ensure adequate power to address the hypothesis being tested?

-Were correct statistical analysis used to support conclusions?

-Are there concerns about ethical or regulatory requirements being met?

Reviewer #2: The authors have successfully addressed my comments regarding the methods they applied, improving their description in the text and acknowledging the limitations.

Reviewer #3: Objectives/hypothesis: The objectives are clear for a synthesis paper, but explicit “testable” claims would benefit from being stated as evidence-tiered conclusions (confirmed/probable/possible occurrences).

Study design: Appropriate (multi-source evidence synthesis + descriptive analyses).

Population described: The “population” is occurrence records; it is described reasonably well, but georeferencing precision and uncertainty treatment need clearer reporting.

Sample size/power: Adequate for the two common species used in encounter analysis; limited for taxa with very few records, requiring cautious inference.

Statistics: The chi-square framework for encounter timing is appropriate for reported encounter counts, provided interpretation remains descriptive and bias-aware.

Ethics/regulatory: No human-subject research is apparent. Ensure permissions/crediting for photographs and privacy/sensitive locality handling are documented.

**********

**Results**

-Does the analysis presented match the analysis plan?

-Are the results clearly and completely presented?

-Are the figures (Tables, Images) of sufficient quality for clarity?

Reviewer #2: The results appropriately match the analysis plan and are presented adequately.

Reviewer #3: The analyses reported generally match the described plan and are clearer than before. To be fully complete and verifiable, results should explicitly link each “confirmed/probable/possible” taxon claim to an evidence tier, and figures/tables should include (or point to) confidence/precision categories for records. Figures appear appropriate and interpretable, but map legends and any encounter figures should remain clearly labeled as reported encounters and remain readable at journal scale.

**********

**Conclusions**

-Are the conclusions supported by the data presented?

-Are the limitations of analysis clearly described?

-Do the authors discuss how these data can be helpful to advance our understanding of the topic under study?

-Is public health relevance addressed?

Reviewer #2: The authors have greatly improved the quality of the conclusions.

Reviewer #3: The conclusions are broadly supported, but must be calibrated by evidence strength (especially for taxa with few records and for antivenom inference). Limitations are now stated more clearly than before; still, they should be consolidated and placed where they directly constrain interpretation (especially for seasonality and public health statements). The paper is helpful for advancing understanding by updating national cobra diversity/distribution and highlighting antivenom testing needs. Public health relevance is clearly addressed, but needs careful conditional wording where efficacy evidence is indirect or absent.

**********

**Editorial and Data Presentation Modifications?**

Use this section for editorial suggestions as well as relatively minor modifications of existing data that would enhance clarity. If the only modifications needed are minor and/or editorial, you may wish to recommend “Minor Revision” or “Accept”.

Reviewer #2: Two minor comments:

1) The authors refer to the markings on the cobras’ hoods as “hood mark”, “hoodmark”, and “hood markings”. For the sake of consistency, I suggest using a single term throughout the text.

2) I would like the authors to ensure that the scientific name of each species is written in full at least the first time it is mentioned in the manuscript.

Reviewer #3: Minor-to-moderate edits would improve clarity: standardize “confirmed/probable/possible” terminology, add a short evidence-tier definition, include a locality-precision field in the dataset, and consider a brief antivenom-evidence summary table/box. Ensure all figures/tables remain legible and self-contained at publication size.

**********

**Summary and General Comments**

Use this section to provide overall comments, discuss strengths/weaknesses of the study, novelty, significance, general execution and scholarship. You may also include additional comments for the author, including concerns about dual publication, research ethics, or publication ethics. If requesting major revision, please articulate the new experiments that are needed.

Reviewer #2: I commend the authors for properly addressing my comments on the original version of the manuscript. I believe this revised version is suitable for publication in PLOS Neglected Tropical Diseases.

Reviewer #3: This is a valuable synthesis with clear relevance to snakebite management. The manuscript is much improved and close to acceptable. Remaining weaknesses are largely about transparency (georeferencing uncertainty) and ensuring that strong claims are reserved for strong evidence. No new experiments are essential; targeted reporting and wording revisions should be sufficient.

**********

PLOS authors have the option to publish the peer review history of their article (what does this mean?). If published, this will include your full peer review and any attached files.

If you choose “no”, your identity will remain anonymous but your review may still be made public.

**Do you want your identity to be public for this peer review?**  For information about this choice, including consent withdrawal, please see our Privacy Policy.

Reviewer #2: No

Reviewer #3: No

[NOTE: If reviewer comments were submitted as an attachment file, they will be attached to this email and accessible via the submission site. Please log into your account, locate the manuscript record, and check for the action link "View Attachments". If this link does not appear, there are no attachment files.] **Figure resubmission:**  While revising your submission, we strongly recommend that you use PLOS’s NAAS tool (https://ngplosjournals.pagemajik.ai/artanalysis) to test your figure files. NAAS can convert your figure files to the TIFF file type and meet basic requirements (such as print size, resolution), or provide you with a report on issues that do not meet our requirements and that NAAS cannot fix.

After uploading your figures to PLOS’s NAAS tool - https://ngplosjournals.pagemajik.ai/artanalysis, NAAS will process the files provided and display the results in the "Uploaded Files" section of the page as the processing is complete. If the uploaded figures meet our requirements (or NAAS is able to fix the files to meet our requirements), the figure will be marked as "fixed" above. If NAAS is unable to fix the files, a red "failed" label will appear above. When NAAS has confirmed that the figure files meet our requirements, please download the file via the download option, and include these NAAS processed figure files when submitting your revised manuscript. **Reproducibility:**  To enhance the reproducibility of your results, we recommend that authors of applicable studies deposit laboratory protocols in protocols.io, where a protocol can be assigned its own identifier (DOI) such that it can be cited independently in the future. Additionally, PLOS ONE offers an option to publish peer-reviewed clinical study protocols. Read more information on sharing protocols at https://plos.org/protocols?utm_medium=editorial-email&utm_source=authorletters&utm_campaign=protocols

Attachment Submitted filename: Naja manuscript comments.docx

# Author comment: Author response to Decision Letter 2

**DOI:** 10.1371/journal.pntd.0014445.r004

4 Jun 2026

Attachment Submitted filename: Response to Reviewers.docx

# Editor report: Decision Letter 2

**DOI:** 10.1371/journal.pntd.0014445.r005

**Authors:** Wuelton Monteiro

5 Jun 2026

Dear Dr. Wüster,

We are pleased to inform you that your manuscript 'The cobras (genus *Naja* ) of Myanmar: An updated species list with information on identification, distributions, and medical importance' has been provisionally accepted for publication in PLOS Neglected Tropical Diseases.

Before your manuscript can be formally accepted you will need to complete some formatting changes, which you will receive in a follow up email. A member of our team will be in touch with a set of requests.

Please note that your manuscript will not be scheduled for publication until you have made the required changes, so a swift response is appreciated.

IMPORTANT: The editorial review process is now complete. PLOS will only permit corrections to spelling, formatting or significant scientific errors from this point onwards. Requests for major changes, or any which affect the scientific understanding of your work, will cause delays to the publication date of your manuscript.

Should you, your institution's press office or the journal office choose to press release your paper, you will automatically be opted out of early publication. We ask that you notify us now if you or your institution is planning to press release the article. All press must be co-ordinated with PLOS.

Thank you again for supporting Open Access publishing; we are looking forward to publishing your work in PLOS Neglected Tropical Diseases.

Best regards,

Wuelton Monteiro, Ph.D.

Section Editor

PLOS Neglected Tropical Diseases

Wuelton Monteiro

Section Editor

PLOS Neglected Tropical Diseases

Shaden Kamhawi

co-Editor-in-Chief

PLOS Neglected Tropical Diseases

orcid.org/0000-0003-4304-636XX

Paul Brindley

co-Editor-in-Chief

PLOS Neglected Tropical Diseases

orcid.org/0000-0003-1765-0002

***********************************************************

# Editor report: Acceptance letter

**DOI:** 10.1371/journal.pntd.0014445.r006

**Authors:** Wuelton Monteiro

Dear Dr. Wüster,

We are delighted to inform you that your manuscript, "The cobras (genus *Naja* ) of Myanmar: An updated species list with information on identification, distributions, and medical importance," has been formally accepted for publication in PLOS Neglected Tropical Diseases.

We have now passed your article onto the PLOS Production Department who will complete the rest of the publication process. All authors will receive a confirmation email upon publication.

The corresponding author will soon be receiving a typeset proof for review, to ensure errors have not been introduced during production. Please review the PDF proof of your manuscript carefully, as this is the last chance to correct any scientific or type-setting errors. Please note that major changes, or those which affect the scientific understanding of the work, will likely cause delays to the publication date of your manuscript. Note: Proofs for Front Matter articles (Editorial, Viewpoint, Symposium, Review, etc...) are generated on a different schedule and may not be made available as quickly.

Soon after your final files are uploaded, the early version of your manuscript will be published online unless you opted out of this process. The date of the early version will be your article's publication date. The final article will be published to the same URL, and all versions of the paper will be accessible to readers.

For Research Articles, you will receive an invoice from PLOS for your publication fee after your manuscript has reached the completed accept phase. If you receive an email requesting payment before acceptance or for any other service, this may be a phishing scheme. Learn how to identify phishing emails and protect your accounts at https://explore.plos.org/phishing.

Thank you again for supporting open-access publishing; we are looking forward to publishing your work in PLOS Neglected Tropical Diseases.

Best regards,

Shaden Kamhawi

co-Editor-in-Chief

PLOS Neglected Tropical Diseases

Paul Brindley

co-Editor-in-Chief

PLOS Neglected Tropical Diseases
